# The spillover effects of fairness events in competitive sports events on audience social trust: a quasi natural experimental analysis

**DOI:** 10.3389/fpsyg.2026.1932641

**Published:** 2026-10-05

**Authors:** Chuanyong Zhang, Meiling Zhou

**Affiliations:** 1 Wuhan University of Bioengineering, Wuhan, Hubei, China; 2 Guangxi Science and Technology Normal University, Laibin, Guangxi, China

**Keywords:** bounded spillover, fairness incidents, social trust, staggered difference-in-differences, surrogate institutional signals

## Abstract

**Introduction:**

How fairness incidents in sports events cross the boundary of entertainment and spill over onto spectators’ social trust has been a continuing controversy in existing research. This paper proposes the framework of “surrogate institutional signals,” which treats the arena, with its explicit rules, public enforcement, and verifiable outcomes, as a low cost diagnostic window on which spectators perform Bayesian updating.

**Methods:**

Merging CFPS and CGSS microdata with Weibo and Zhihu text streams and embedded trust game experiments, the study screens 486 candidate incidents by independent double coding into a database of 63 fairness incidents covering 2018 to the first half of 2025, distinguishes the incidents into regulative, procedural, and substantive types, and measures trust as a ladder of six tiers ordered by institutional distance from the focal incident. Identification combines the Callaway-Sant’Anna staggered difference-in-differences weighted by attention with synthetic control, and adds competing mediation and causal forest analysis.

**Results:**

The core results show that the absolute value of the negative spillover effect of procedural incidents on social trust is 3.29 times that of regulative incidents and 1.88 times that of substantive incidents; the effect attenuates monotonically along the trust ladder, from −0.612 for the officiating crew and organizing committee to −0.221 for public institutions outside sport, or 36.1% of the innermost effect, and to −0.129 for generalized trust in strangers, while close ties show no detectable change and 67.0% of the outside sport effect survives conditioning on dissatisfaction with the incident; the surrogate institutional signal pathway carries 51.3% of the spillover effect, while moral shock and media accessibility contribute 28.4 and 20.3% respectively, and the three pathways display the temporal evolution of “emotion first, cognition takes over”; conditional average treatment effects are significantly heterogeneous, with the subgroup high in both political interest and media use responding most strongly.

**Discussion:**

The study identifies a bounded and graded rather than a diffuse spillover, delimits the reach of trust updating across domains, and provides actionable mechanistic evidence for event governance and information disclosure.

## 1 Introduction

Competitive sports events are among the most mobilizing public events in modern society: hundreds of millions of spectators watch simultaneously through stadiums, screens, and social platforms, sharing one set of refereeing rules and one set of outcomes, which makes the arena a highly observable window through which the public examines social fairness. Match fixing, doping violations, refereeing disputes, and league anticorruption cases have been intensively exposed worldwide in recent years; between 2022 and 2025 investigations against gambling and corruption swept Chinese football and dozens of former national team players and officials received lifetime bans, and such cases now occupy trending lists and mainstream headlines rather than sports sections alone. When spectators witness the public violation of arena rules, whether the cognitive impact stops at disappointment with the events themselves or transmits further to trust judgments about institutions beyond the arena constitutes an important question that has not yet received rigorous causal identification. The question is posed at three separable levels rather than as a single leap from one incident to society: trust in the actors that produced the disputed outcome, namely the officiating crew and the organizing committee; trust in the bodies that own the violated rules, namely the league, the national single sport association, and the sport administration system supervising them; and trust in public institutions outside sport, measured as courts, local administrative departments, and public service institutions, alongside trust in strangers and in close ties. Political legitimacy and regime support are neither measured nor claimed here, and the term spillover is reserved for movement from the first two levels to the third. Social trust underpins cooperation, transactions, and governance efficiency, so exogenous shocks from high attention public events deserve serious examination.

The existing literature approaches this question from two ends without joining them. Sport management research keeps outcome variables inside the event: information about sport corruption weakens trust in sport organizations and communities (Manoli et al., 2024), fans of different types update attitudes differently after scandals (Sun et al., 2023), public trust in elite sport institutions is driven jointly by event and governance performance (Funahashi and Zheng, 2023), and doping incidents reduce trust in event fairness without visibly suppressing viewing demand (Otto et al., 2021). Trust research enters from the other end but takes political and economic crises rather than sport as the shock source: institutional quality causally raises generalized trust in laboratory tests (Martinangeli et al., 2024), cross-lagged analysis identifies the dynamic structure linking institutional and social trust (Glatz and Schwerdtfeger, 2022), and six country panel evidence shows political trust fluctuating sharply after scandals before returning to equilibrium (Devine and Valgarðsson, 2024). Chinese evidence is equally scattered, covering the short run suppression of public trust and perceived fairness by a public health shock (Wei et al., 2022) and the chain from governance quality to wellbeing via perceived fairness and trust in government (Ma et al., 2024b). Neither strand asks whether fairness incidents in sports events transmit across domains to social trust.

Addressing these gaps, this study takes “surrogate institutional signals” as its explanatory framework and identifies the spillover effects of fairness incidents on spectators’ social trust. Publicly verifiable institutional samples are scarce, whereas the arena, with explicit rules, public enforcement, and verifiable outcomes, is a diagnostic window available at low cost; where existing work summarizes trust shocks as emotional contagion or identity categorization, this study reconstructs them as inference across domains in which spectators update Bayesian beliefs about institutional credibility. The paper decomposes fairness incidents into three categories, regulative, procedural, and substantive, and decomposes trust into a ladder of six tiers ordered by institutional distance, running from the officiating crew and the organizing committee, through the league and national single sport association, the sport administration system, and public institutions outside sport, to generalized trust in strangers and trust in close ties. The resulting 3 × 6 spillover matrix turns the reach of the spillover into an object of measurement rather than an assumption, and responds to the type heterogeneity obscured when existing research renders both dimensions unidimensional (Manoli et al., 2024; Funahashi and Zheng, 2023). On the data side it fuses the China Family Panel Studies and the Chinese General Social Survey with Weibo and Zhihu text sentiment and behavioral data from embedded trust games, cross validating three sources against doubts about the measurement validity of subjective trust items (Glatz and Schwerdtfeger, 2022). Identification builds exogenous shocks from the incidents intensively exposed in mainland China from 2018 to the first half of 2025, joins the Callaway-Sant’Anna staggered difference-in-differences with synthetic control, and weights continuous treatment intensity by news volume and search indices, which addresses treatment group contamination and violations of parallel trends (Devine and Valgarðsson, 2024; Wei et al., 2022). Mechanism identification specifies three competing pathways, institutional signals, moral shock, and media accessibility, instruments the accessibility channel with exogenous media coverage shocks, and uses the causal forest to locate the spectator subgroups most sensitive to the treatment effect (Martinangeli et al., 2024; Ma et al., 2024b). The three mediators are treated as measured constructs rather than labels: each is given an explicit item set, assessed for internal consistency and discriminant validity, and required to predict trust at the tiers beyond the arena once dissatisfaction with the focal incident is held constant, which is the condition under which a mediator carries generalization rather than event specific affect.

## 2 Literature review and theoretical construction

### 2.1 The typological bottleneck: unidimensional treatment of fairness incidents and trust dimensions

Most existing analyses of fairness incidents adopt binarized or unidimensional treatments. A global data study using the Macolin Convention typology separates competition manipulation into direct interference, identity modification, and rule violation, and finds significant differences across categories in regional distribution and annual trends (Lavallee et al., 2026). A survey of Chinese Super League fans shows that league governance failure and commercialization form dual dimensions of fan attitudes that a single support versus opposition split cannot capture (Ma et al., 2024a), a United Kingdom experiment reports markedly differentiated responses to corruption information across spectator backgrounds (Manoli et al., 2024), and analysis of scandal responses makes a preliminary crossing of fan typology with incident nature (Sun et al., 2023). Trust dimensions are treated just as simply: recent measurement work replaces the single item measure of generalized trust with the Stranger Face Trust and Imaginary Stranger Trust scales and confirms a multidimensional structure interwoven from concrete trust targets and situations (Robbins, 2023), while behavioral validation on Chinese samples finds clearly low correlations between trust games and survey items for ingroup and outgroup trust (Tang and Gong, 2024). What the double simplification obscures is not only the number of trust dimensions but their ordering. Trust targets differ in institutional distance from the observed incident, and an index that pools an officiating crew, a national association, and a court into one score cannot show how far an incident travels, so the reach of any reported spillover remains indeterminate. It is also an important source of unstable effect estimates and divergent conclusions in spillover research.

### 2.2 The causal identification bottleneck: methodological limits of counterfactual construction with observational data

Observational research on these incidents faces strict obstacles to causal identification: incidents are not randomly assigned, treatment timing is staggered, and the estimation weights of two-way fixed effects models can turn negative, so the estimator deviates from the target ATT. Methodological work shows that standard staggered difference-in-differences produces bad comparisons under heterogeneous treatment effects and that the group-time average treatment effect is the reliable estimand (Callaway and Sant’Anna, 2021); replications in finance show that ignoring the problem biases estimates and can even reverse signs (Baker et al., 2022); and decomposition results show that TWFE estimates under timing heterogeneity cannot be mapped uniquely onto causal estimands (Goodman-Bacon, 2021). Synthetic control offers a complementary path through weighted counterfactuals (Abadie, 2021), and causal forests identify conditional average treatment effects robustly under multiple moderators (Zheng and Yin, 2023). Experimental evidence on institutional trust further shows that identifying an association without mechanism pathways conceals the working channel from institutional quality to trust updating (Martinangeli et al., 2024). Existing research on sport shocks remains at before and after comparisons or simple difference-in-differences, which is the main methodological source of inconsistent conclusions.

### 2.3 The mechanism black box bottleneck: the missing identification of competing transmission pathways

Systematic testing of the intermediate mechanisms is still missing. Moral emotion is one candidate: moral outrage is amplified through social learning, and observers overestimate the outrage of others, which inflates beliefs about intergroup hostility (Brady et al., 2023); third party punishers who express emotion are trusted more than those who impose only financial punishment, so moral emotion itself signals credibility (Kupfer and Tybur, 2023); and the severity and deservedness of punishment moderate the strength of that signal (Salcedo and Jimenez-Leal, 2024). Institutional performance is a second candidate, since policy performance affected political trust in the early COVID-19 period (Belchior and Teixeira, 2023) and crisis exposure suppressed interpersonal trust lastingly in Chinese microdata, a social scar mechanism (Fang et al., 2023). Information accessibility is a third, because coverage intensity conditions the moderating effect of threat perception (De León et al., 2023), while cross-lagged evidence on the dynamic structure between institutional and social trust indicates that mediation identification must rest on bidirectional feedback (Glatz and Schwerdtfeger, 2022). Tests of the three pathways remain separate, and their relative contributions have not been compared within one framework.

### 2.4 Introducing the surrogate signal theory and research hypotheses

Responding to these bottlenecks requires a framework that accommodates type heterogeneity, rigorous identification, and mechanism decomposition at once. The framework of “surrogate institutional signals” rests on a basic observation: publicly verifiable institutional samples on which spectators can rely are extremely scarce, whereas the regulated character, openness, and outcome verifiability of the arena make it a unique diagnostic window available at low cost. When spectators witness rules within competition being publicly violated, they obtain an inferential sample about how rules are enforced, and they update trust in a Bayesian manner with a precision that declines as the trust target moves away from the arena. The framework therefore predicts a graded rather than a uniform response: updating is strongest for the bodies that enforce the violated rules, weaker for institutions that share with the arena only the general property of enforcing rules, and absent for concrete personal ties, which rest on direct experience rather than on institutional inference. Existing evidence supports the temporal and credibility dimensions of this logic: information quality buffers the negative impact of event shocks on political trust in the Shanghai lockdown (Zhai and Han, 2024), the negative effect of major public shocks on social trust exists but is transient in South Korean surveys from 2016 to 2023 (Kim, 2025), Dutch panel data show institutional trust to be stable in adulthood while allowing brief fluctuations during shocks (la Roi et al., 2025), and six country panel evidence reports short run fluctuation after scandals followed by a return to equilibrium (Devine and Valgarðsson, 2024). Following this logic, the paper proposes three groups of core research hypotheses comprising four testable propositions. H1a: Fairness incidents in sports events exert negative spillovers on spectators’ social trust; the effect peaks within 6 to 12 months after incident exposure and gradually decays after 24 months. H1b: The spillover is bounded and graded rather than uniform; the absolute effect declines monotonically along the trust ladder as institutional distance from the focal incident increases, and trust in close ties shows no detectable change. H2: Spillover effects differ systematically across incident types; because procedural incidents expose the institutional diagnostic signal that “rules can be systematically manipulated,” their spillover strength is significantly higher than that of regulative and substantive incidents (Funahashi and Zheng, 2023). H3: Surrogate institutional signals occupy the dominant position among transmission pathways, moral shock and media accessibility constitute secondary pathways, and effects are systematically heterogeneous across spectator subgroups, especially along political attention and media use.

## 3 Research design

### 3.1 Typological screening of the three categories of fairness incidents and construction of the quasi natural experiment

Typological screening is the preliminary stage of the design. Along the institutional signal dimension, incidents fall into three categories: regulative incidents, disputes over the interpretation and enforcement of rules within competition, typified by public challenges to refereeing decisions; procedural incidents, systematic manipulation violating competition procedures, represented by match fixing, collective deliberate underperformance, and illicit benefit transfers; and substantive incidents, violations of substance and bodily rules, with doping at the core. The three differ qualitatively in the strength of the institutional signal available to spectators, which is why they are treated separately.

An incident enters the database only if it simultaneously satisfies three preconditions: official agencies intervene to investigate or publicly characterize the case, mainstream media reports within 1 week exceed the specified threshold, and the daily Baidu search index B shows a marked peak in the same period. Taking the anticorruption investigation of the Chinese Super League (CSL) between 2022 and 2024 as the core source and also covering public incidents in the CBA, track and field, and swimming, the study builds an incident database covering 2018 to the first half of 2025. Screening of the 486 candidate incidents retains 63 that satisfy all three preconditions, comprising 27 regulative, 19 procedural, and 17 substantive incidents, while 423 are excluded for failing a precondition or falling outside the three categories. The screening decision process is shown in Figure 1.

**Figure 1:**
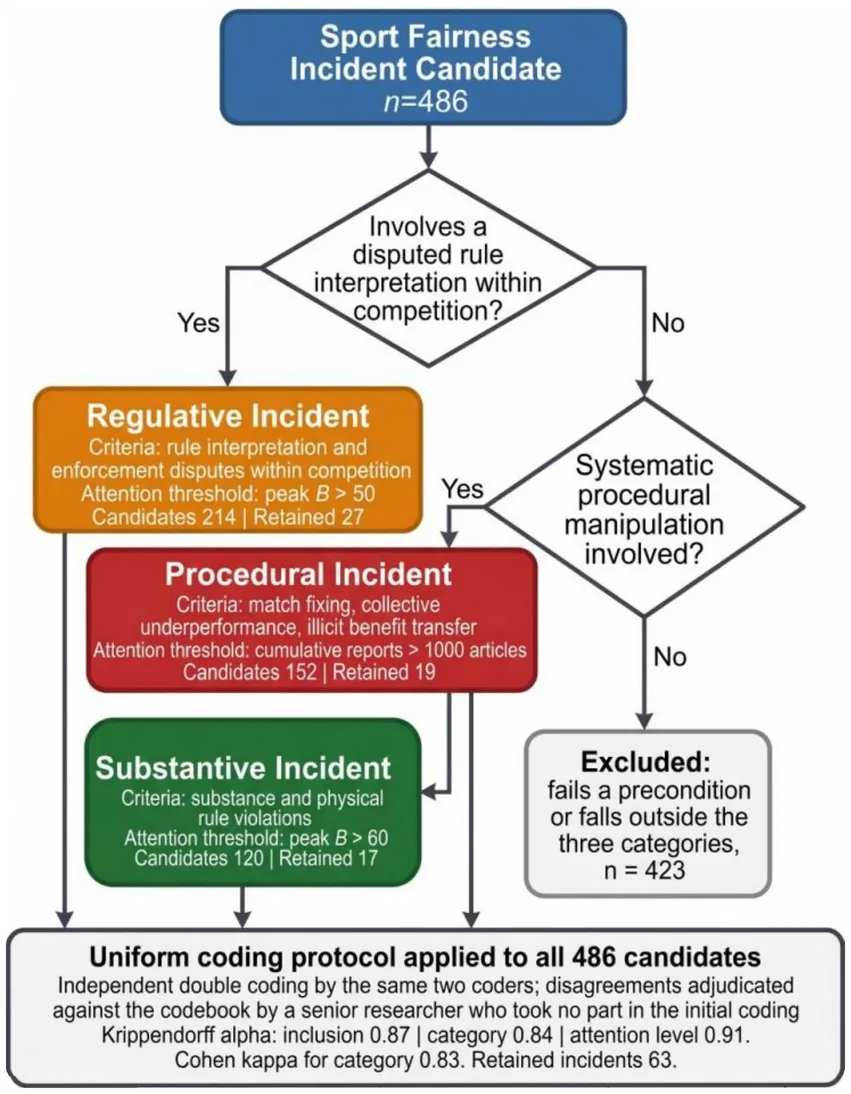
Decision tree for the typological screening of the three categories of fairness incidents.

The temporal distribution of incidents and differences in their intensity are the identifiable sources of the staggered design, and are shown in Figure 2: procedural incidents peak markedly between 2022 and 2024, whereas substantive incidents are sparse but individually impactful. The identification criteria and coding rules are shown in Table 1.

**Figure 2:**
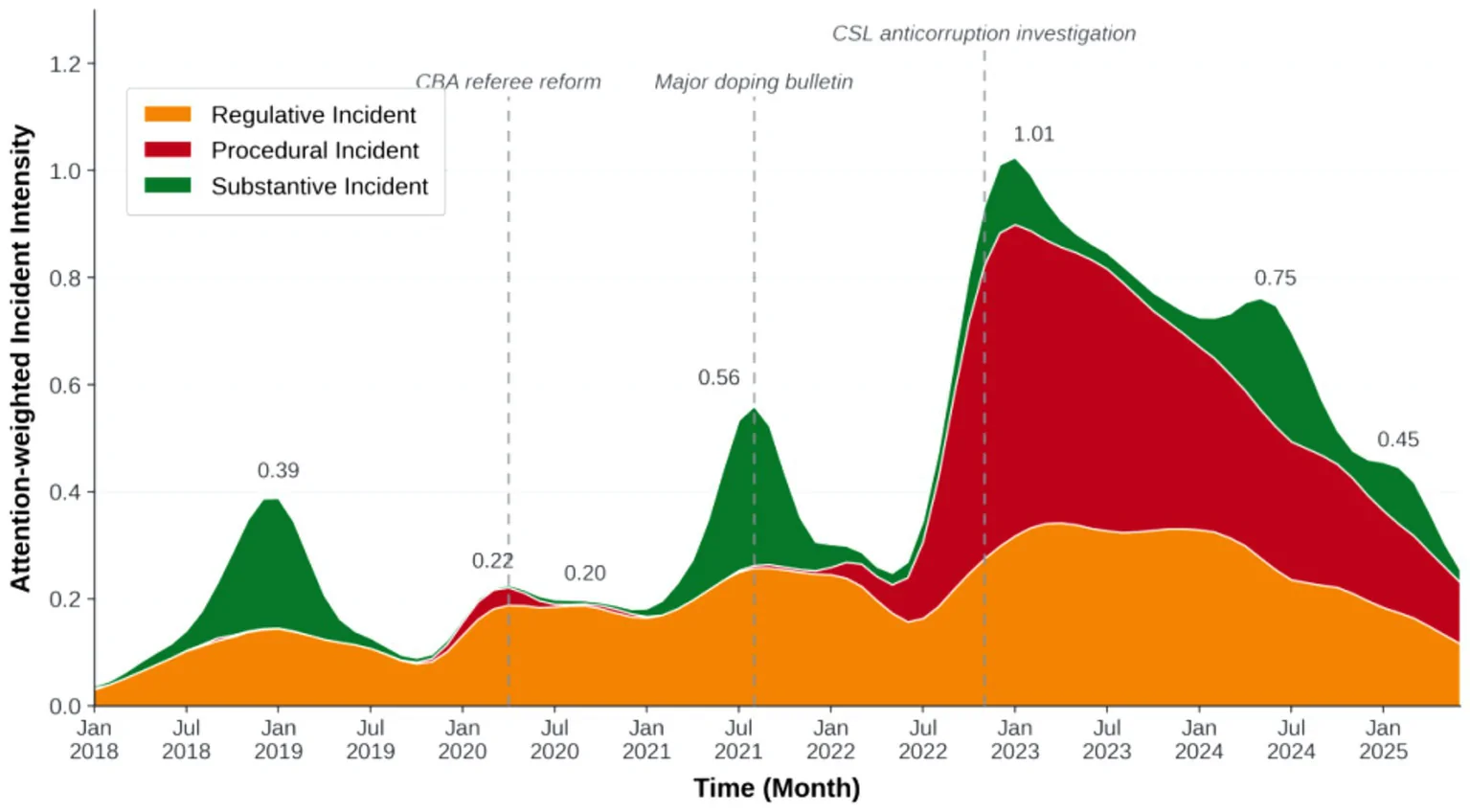
Temporal distribution of major fairness incidents in Chinese sports events from 2018 to the first half of 2025.

**Table 1:** Typological identification criteria, coding rules, and intercoder reliability for the three categories of fairness incidents.

Incident category	Core identification criteria	Exclusion conditions	Attention threshold	Candidate units	Retained incidents	Adjudicated disagreements	Percentage agreement (%)	Krippendorff alpha
Regulative incidents	Disputes over rule interpretation and enforcement within competition	Purely technical operational errors	Peak B >50	214	27	18	91.6	0.84
Procedural incidents	Systematic procedural manipulation and illicit benefit transfers	Isolated disputes without official characterization	Cumulative reports >1,000 articles	152	19	9	94.1	0.90
Substantive incidents	Violations of substance and bodily rules	Cases without official characterization	Peak B >60	120	17	10	91.7	0.87

The adjudicated disagreements, percentage agreement, and Krippendorff alpha in each row refer to the inclusion decision within that category’s candidate stream. Across all 486 candidates the inclusion decision reached 92.4% agreement with an alpha of 0.87 and 37 adjudications; the category assignment of the 63 retained incidents reached 88.9% with an alpha of 0.84 and a Cohen kappa of 0.83, requiring 7 adjudications; the ordinal attention level reached an alpha of 0.91.

The coding protocol is uniform across the three categories and the same two trained coders applied it to every candidate incident. A written codebook fixes the operational rule for each precondition: official characterization is coded as present only when an agency holding disciplinary or judicial authority issues a named decision, an investigation notice, or a sanction list, so press summaries without an identifiable issuing agency do not qualify; report volume is counted from three mainstream media databases over the 7 days following first exposure; and the search peak is coded from the daily Baidu index against the median of the preceding 90 days. The two coders first completed a pilot round on 60 units drawn from a separate 2016 to 2017 screening window that is not part of the analysis sample, and were required to reach a Krippendorff alpha of at least 0.80 on every dimension before the main round, and none of the units used for training entered the analysis sample. They then coded all 486 candidate incidents independently on the inclusion decision, the category assignment, and the attention level, without access to each other’s judgments, and disagreements were resolved by a senior researcher who took no part in the initial coding and adjudicated against the codebook rather than through discussion, so agreement was not produced by negotiation. Agreement on the inclusion decision reached 92.4% across the 486 candidates with a Krippendorff alpha of 0.87; the category assignment of the 63 retained incidents reached 88.9% with a Krippendorff alpha of 0.84 and a Cohen kappa of 0.83; the ordinal attention level reached a Krippendorff alpha of 0.91. Adjudication was required for 37 of the 486 inclusion decisions and for 7 of the 63 category assignments. Because percentage agreement does not correct for chance and overstates reliability for nominal variables, the chance corrected coefficients are reported alongside it, and reliability by category stream is shown in Table 1.

The incident intensity indicators provide a direct basis for the subsequent weighting of treatment intensity. The construction principles of the quasi natural experiment above also draw on the identification norms for local treatment effects under the LATE framework (Angrist, 2022).

### 3.2 Multisource data fusion of survey microdata, social media texts, and trust games

Trust measurement is methodologically dispersed, so the fusion design connects measures of different granularity to one incident exposure timeline. CFPS and CGSS micro panel data provide the longitudinal baseline, Weibo and Zhihu text captures emotional reactions and moral framing during exposure, and embedded trust games provide behavioral measures before and after events, correcting social desirability bias. The three sources do not measure the same trust target, and the division of labor among them is what makes the reach of the spillover estimable. The survey microdata carry the tiers outside sport, namely institutional trust, generalized trust in strangers, and trust in close ties. The embedded panel carries the tiers inside sport, namely the officiating crew and organizing committee, the league and national single sport association, and the sport administration system, none of which appears in general purpose survey instruments. Each panel wave also repeats the outside sport items, so the two sources overlap there and the whole ladder sits on one metric. The fusion architecture is shown in Figure 3, with the exposure timeline as the connecting spine and strict timestamp alignment as the key design condition.

**Figure 3:**
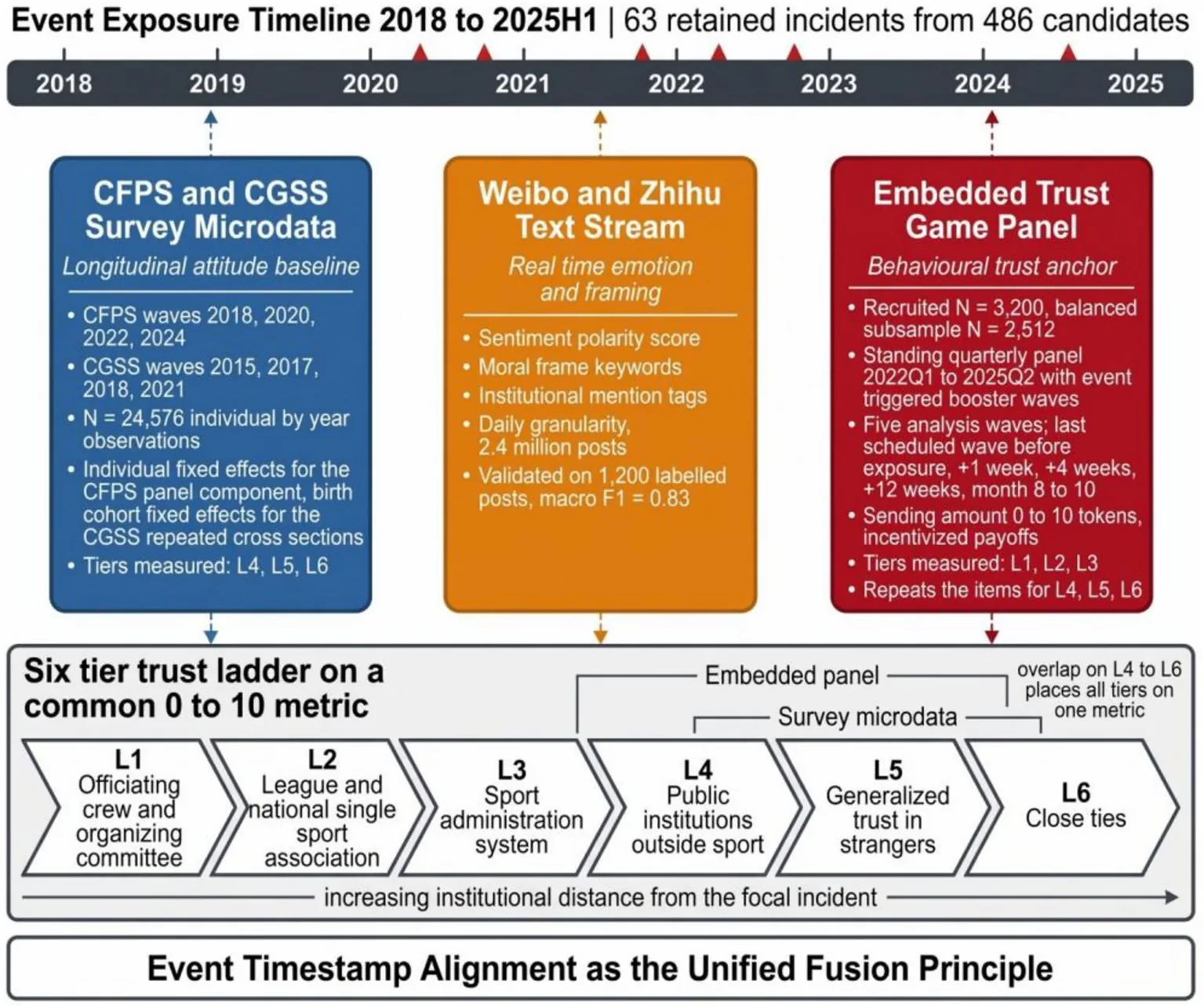
Architecture of the fusion of the three data sources: survey microdata, social media texts, and trust games.

Text processing follows a framework tailored to social science contexts (Hassan et al., 2025), building incident related subsets from keywords and applying three parallel layers to a corpus of 2.4 million posts collected at daily granularity: sentiment polarity, moral framing, and institutional mention tagging. For the behavioral games, an embedded design combines a standing quarterly schedule with event triggered booster waves. The panel is fielded every quarter from the first quarter of 2022 to the second quarter of 2025, so that every respondent is measured at least once before the first incident that reaches them, and three booster waves are added at 1 week, 4 weeks, and 12 weeks after an incident is officially characterized. Five measurement points per respondent enter the analysis: the last scheduled wave before exposure, the three booster waves, and the scheduled wave that falls between month 8 and month 10 after exposure, so that game decisions and exposure intensity form a controllable temporal relationship. Because exposure timing cannot be anticipated, the pre exposure measurement comes from the standing schedule rather than from a wave designed around a particular incident, and respondents not yet exposed serve as the comparison group under the same not yet treated logic used in the main analysis. The last post exposure measurement falls inside the 6 to 12 month window that Hypothesis H1a specifies, so the panel spans both the early emotional period and the later cognitive one. The design therefore supplies trust signals at three levels, attitude, emotion, and behavior, under one incident identification framework. The panel comprises N = 3,200 individuals recruited through an online research panel stratified by city, age, and gender, with completion above 78% across the five analysis waves.

Trust is measured as a ladder of six tiers ordered by institutional distance from the focal incident, all rescaled to a common 0 to 10 metric. The ordering is the object under test, since generalized trust has been shown to be woven from multiple concrete trust targets rather than to constitute a single dimension (Robbins, 2023), and survey items and behavioral games diverge systematically across trust targets in Chinese samples (Tang and Gong, 2024). The inner tiers separate targets that existing work tends to merge: the referee crew and organizing committee of the disputed match are kept apart from the league and national single sport association, and both from the sport administration system that supervises them. The generalized social trust index used in the baseline analysis is retained unchanged as the headline outcome, and the ladder is estimated alongside it rather than in place of it. Item wording is held constant across waves, and the survey tiers keep the established wording of the two national instruments so that estimates remain comparable with existing evidence. The tiers estimated on the embedded panel use the balanced subsample of N = 2,512 individuals who completed all five analysis waves, yielding 12,560 individual by wave observations. Internal consistency for the tiers measured by several items ranges from 0.81 to 0.88, and a confirmatory factor analysis supports the separation of the tiers, with the highest correlation between adjacent tiers reaching 0.63 and no correlation between non adjacent tiers exceeding 0.48. The definitions, item content, sources, and reliability of the six tiers are shown in Table 2.

**Table 2:** Definitions, item content, sources, and reliability of the six trust tiers.

Tier	Trust target	Item content	Number of items	Data source	Cronbach alpha
L1	Officiating crew and organizing committee of the focal incident	Referee crew, match commissioner and organizing committee, and the panel handling the case	3	Embedded panel	0.88
L2	League and national single sport association	League company, national single sport association, and their disciplinary and appeal procedures	3	Embedded panel	0.85
L3	Sport administration system as a whole	Sport administration department at national and provincial level	2	Embedded panel	0.81
L4	Public institutions outside sport	Courts, local administrative departments, and public service institutions of the respondent’s city	3	CFPS, CGSS, embedded panel	0.83
L5	Generalized trust in strangers	Most people the respondent does not know personally	1	CFPS, CGSS, embedded panel	—
L6	Close ties	Respondent’s relatives and close friends	1	CFPS, CGSS, embedded panel	—

All tiers are rescaled to a 0 to 10 metric. Single item tiers follow the established survey wording, for which no alpha is reported. The trust game transfer share serves as an external anchor for behavioral validity and is not one of the six tiers.

The three mediators are measured as constructs rather than treated as labels. Perceived institutional signal strength is measured by four items on whether the incident indicates that rules can be circumvented, that enforcement is selective, that supervision is ineffective, and that comparable conduct is widespread. Moral shock is measured by three items on anger, indignation, and perceived betrayal, and is cross validated against the text based sentiment index, which was itself validated on 1,200 manually labelled posts and reaches a macro F1 of 0.83 against human labels whose own Krippendorff alpha is 0.85. Media accessibility is measured by three indicators covering exposure frequency, platform breadth, and self reported ease of following the case. Composite reliability ranges from 0.82 to 0.89 and the average variance extracted from 0.61 to 0.68, the confirmatory model fits the data well, and the heterotrait monotrait ratios among the three constructs remain below the 0.85 criterion, so the mediators are empirically distinguishable rather than three labels for one reaction. Because a mediator that registers only dissatisfaction with the focal incident cannot carry generalization, each construct is additionally required to predict trust at the tiers beyond the arena, and that requirement enters the identification design as an explicit condition rather than an interpretive claim. Measurement and validity evidence is shown in Table 3.

**Table 3:** Measurement and validity of the three competing mediators.

Mediator	Items or indicators	Cronbach alpha	Composite reliability	AVE	HTMT with institutional signal	HTMT with moral shock
Perceived institutional signal strength	4	0.87	0.89	0.67	—	0.61
Moral shock	3	0.84	0.86	0.68	0.61	—
Media accessibility	3	0.79	0.82	0.61	0.44	0.39

Confirmatory factor analysis on the embedded panel yields $\chi^{2}$ /df = 2.41, CFI = 0.968, TLI = 0.958, RMSEA = 0.038, and SRMR = 0.034. All heterotrait monotrait ratios fall below the 0.85 criterion.

### 3.3 Joint identification strategy combining staggered DID weighted by attention and synthetic control

Treatment timing is systematically staggered and conventional two-way fixed effects models can hardly recover the target ATT, so identification takes the attention weighted staggered difference-in-differences as its main framework, with the Callaway-Sant’Anna group-time average treatment effect as the estimand. The weighting step embeds report intensity and search indices into the weight structure so that intense incidents are not diluted under equal weight aggregation, as shown in Equation 1.

$$\tau_{\mathrm{ATT}}^{w}=\sum_{g\in G}\sum_{t\geq g}w_{g,t}\cdot \mathrm{ATT}(g,t)$$ (1)

where $\tau_{\mathrm{ATT}}^{w}$ denotes the total average treatment effect weighted by attention; G denotes the set of all exposed groups; g denotes the exposed group index; t denotes the time index; $w_{g,t}$ denotes the group-time attention weight; ATT(g,t) denotes the unweighted group-time average treatment effect.

The attention indicator combines media report volume and the intensity of users’ active searching, with a logarithmic transformation to mitigate the heavy tail of the distribution, as shown in Equation 2.

$$A_{g,t}=\alpha \log(1+N_{g,t})+(1-\alpha )\log(1+B_{g,t})$$ (2)

where $A_{g,t}$ denotes the group-time attention intensity; $N_{g,t}$ denotes the number of mainstream media reports on the incident of group g at time t ; $B_{g,t}$ denotes the Baidu search index at time t for the incident of group g ; α denotes the mixing parameter between reporting and searching, ranging from 0 to 1 with a baseline setting of 0.5. The weight $w_{g,t}$ in Equation 1 equals $A_{g,t}$ divided by its sum over all exposed group-time cells in the sample, which guarantees that the weights sum to 1 while preserving relative intensity information.

Synthetic control is added as an independent robustness check. Because the sample of procedural incidents is small and the precision of the staggered DID is limited, it supplies a counterfactual independent of DID by constructing the weighted combination closest to the exposed units before the events. Its constrained optimization is shown in Equation 3.

$$\min_{w}∥X_{1}-X_{0}w{∥}_{V},s.t.w\geq 0,1^{\prime }w=1$$ (3)

where w denotes the weight vector of the control units; $X_{1}$ denotes the vector of characteristics of the exposed unit before the events; $X_{0}$ denotes the matrix of characteristics of the control unit pool before the events; V denotes the diagonal covariate weighting matrix; 1 denotes a vector of ones of conformable dimension. For recent methodological details of synthetic control, see the relevant review (Gilchrist et al., 2023).

The joint identification strategy is shown in Figure 4: DID provides the main analytical framework, synthetic control provides independent comparisons for rare incidents, and the two cross check the sign, direction, and significance of the effects.

**Figure 4:**
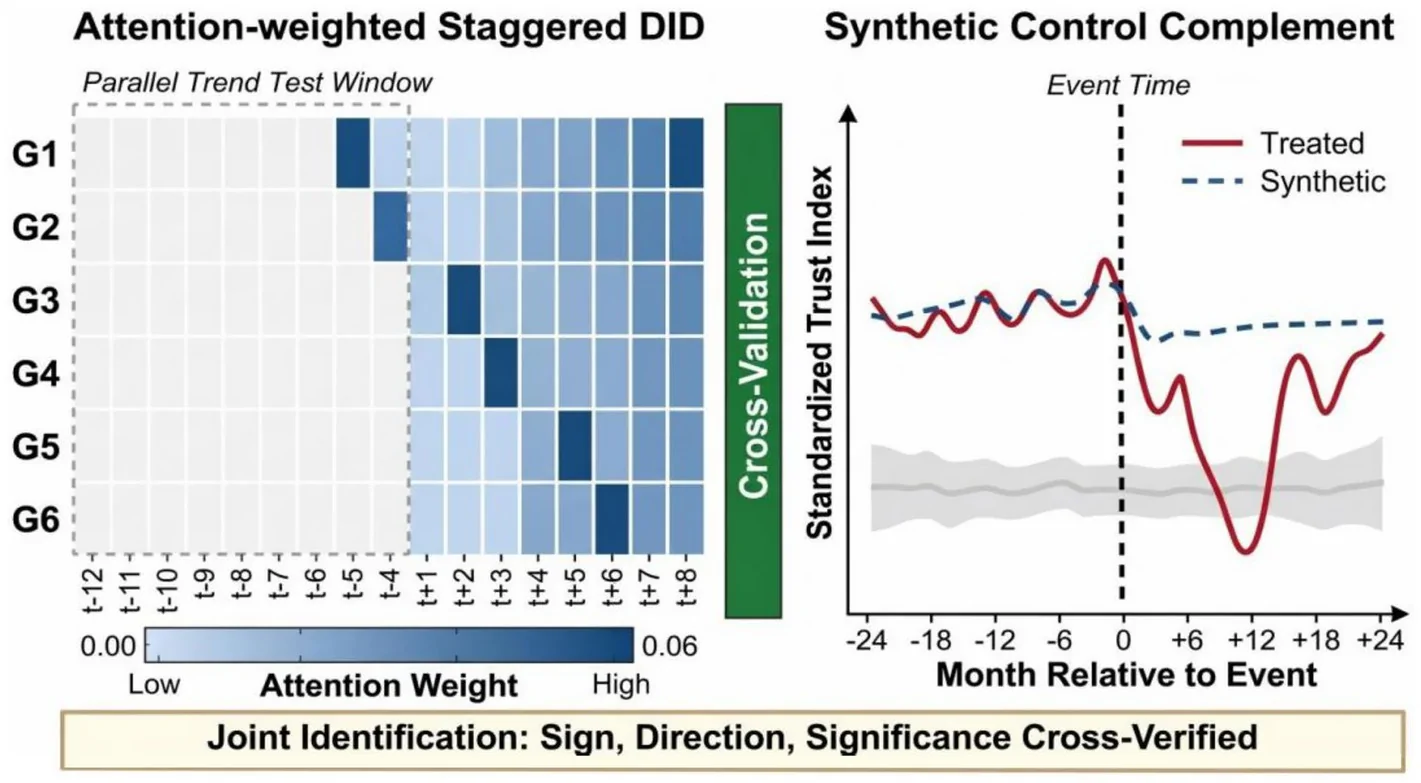
Schematic of the joint identification strategy combining staggered DID weighted by attention and synthetic control.

### 3.4 Competing mechanism decomposition and the causal forest heterogeneity identification framework

Because the three candidate pathways of surrogate institutional signals, moral shock, and media accessibility coexist, a single mediator model cannot deliver accurate relative contributions, so the competing mediation framework enters all three simultaneously and instruments pathways with potential endogeneity. The instrument for media accessibility is the interaction between regional internet infrastructure density and the policy regulation window at exposure, correlated with the intensity of individual perception while satisfying the exclusion restriction with respect to the other two mediators. The core estimation is shown in Equation 4.

$$\begin{array}{l} \text{ΔTrus}t_{i,t}=\alpha +\beta_{0}D_{i,t}+\beta_{1}M_{i,t}^{\mathrm{sig}}+\beta_{2}M_{i,t}^{\mathrm{emo}}\\ +\beta_{3}{\hat{M}}_{i,t}^{\mathrm{med}}+\gamma^{\prime }X_{i,t}+\varepsilon_{i,t} \end{array}$$ (4)

where $\text{ΔTrus}t_{i,t}$ denotes the trust change of individual i at time t ; $D_{i,t}$ denotes the incident exposure indicator; $M_{i,t}^{\mathrm{sig}}$ denotes the institutional signal intensity indicator; $M_{i,t}^{\mathrm{emo}}$ denotes the moral shock intensity constructed from text sentiment indicators; ${\hat{M}}_{i,t}^{\mathrm{med}}$ denotes the fitted value of media accessibility from the instrumental variable; $X_{i,t}$ denotes the vector of control variables; γ denotes the vector of regression coefficients on the control variables; $\varepsilon_{i,t}$ denotes the disturbance term.

The directed acyclic graph is shown in Figure 5: the three pathways feed into trust change in parallel, the instrument enters only through the media pathway, and surrogate institutional signals are designated dominant, with weight determined by the data. The outcome node is the six tiers of the trust ladder rather than a single trust change, and dissatisfaction with the focal incident enters as a separate node pointing to every tier, separating the component travelling with event specific affect from the component transmitted by institutional inference.

**Figure 5:**
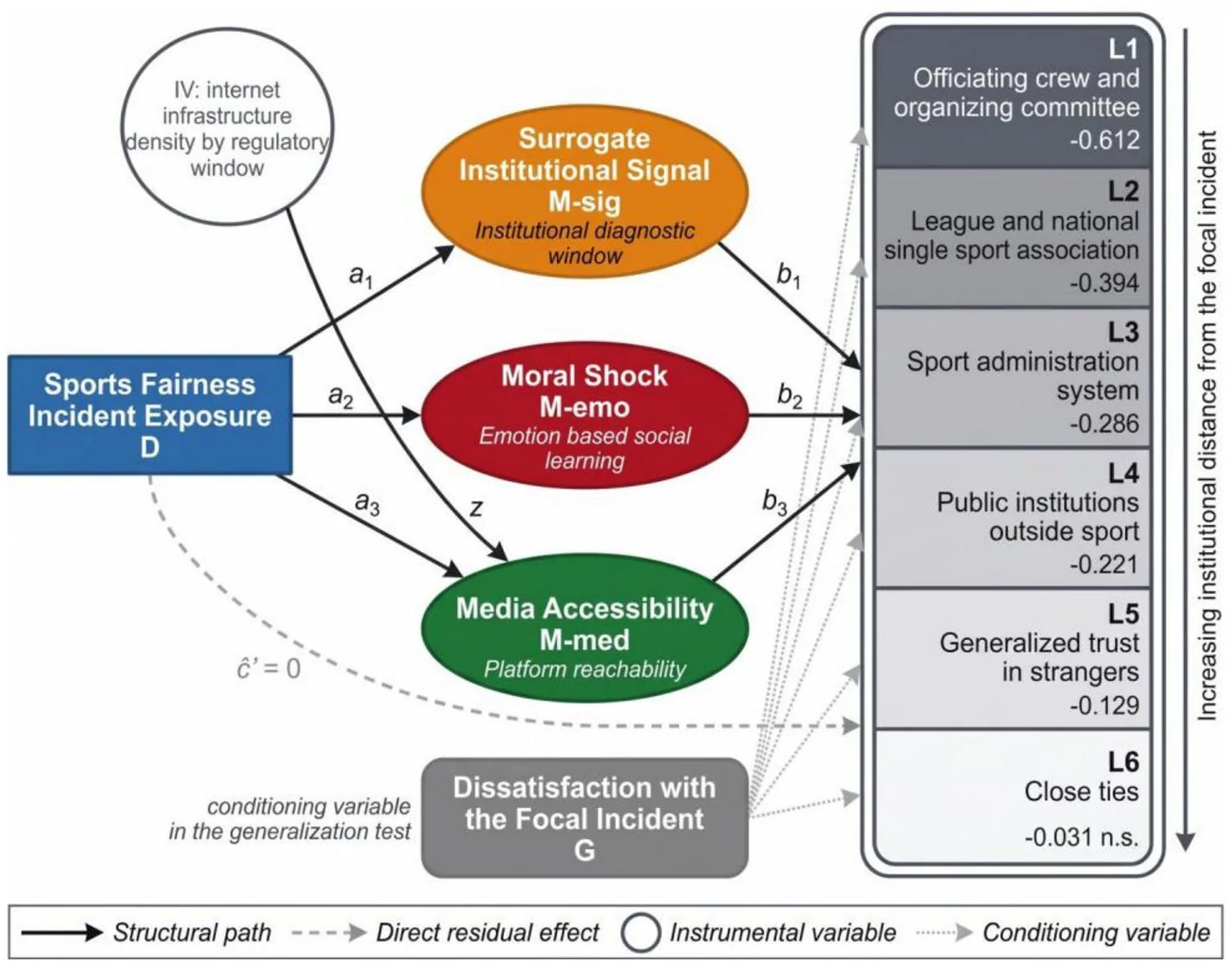
Directed acyclic graph (DAG) of the competing mechanism pathways.

Decomposing an effect into pathways rests on assumptions estimation alone cannot verify, in particular the absence of unmeasured confounding between each mediator and the outcome, so the identification claim is stated and then probed rather than asserted (Qin, 2024). Two additional requirements are imposed. First, each mediator must predict trust at the tiers beyond the arena once dissatisfaction with the focal incident is held constant, because a mediator registering only event specific affect cannot carry the claimed generalization. Second, the outer tier estimates are re estimated with that dissatisfaction and its interaction with exposure as controls. Because dissatisfaction is realized after exposure, conditioning on it does not yield a causal decomposition; the conditional estimate is read as a conservative lower bound and the unconditional estimate as its upper bound. The specification is shown in Equation 5.

$$\Delta T_{i,t}^{\left(\ell \right)}=\beta_{0}+\beta_{1}D_{i,t}+\beta_{2}D_{i,t}G_{i,t}+\beta_{3}G_{i,t}+\gamma^{\prime }X_{i,t}+\mu_{i}+\lambda_{t}+\varepsilon_{i,t}$$ (5)

where $\Delta T^{\left(\ell \right)}$ denotes the trust change of individual i at time t at ladder tier ℓ ; D denotes attention weighted incident exposure; G denotes dissatisfaction at the level of the focal incident; X denotes the control vector and γ its coefficient vector; μ and λ denote individual and time fixed effects; ε denotes the disturbance term. The coefficient $\beta_{1}$ surviving conditioning on G at the outer tiers is the quantity bearing on generalization.

Spectator subgroups differ systematically in interpreting incidents, so a uniform effect would conceal distributional information of policy value. The causal forest robustly estimates conditional average treatment effects under multiple covariates (Murakami et al., 2022), here age, education, political interest, type of daily media use, urbanization level, and engagement with incident topics; its execution and results are shown in Figure 6. Typological screening, multisource fusion, joint identification, and the causal forest together respond to the triple bottlenecks set out above.

**Figure 6:**
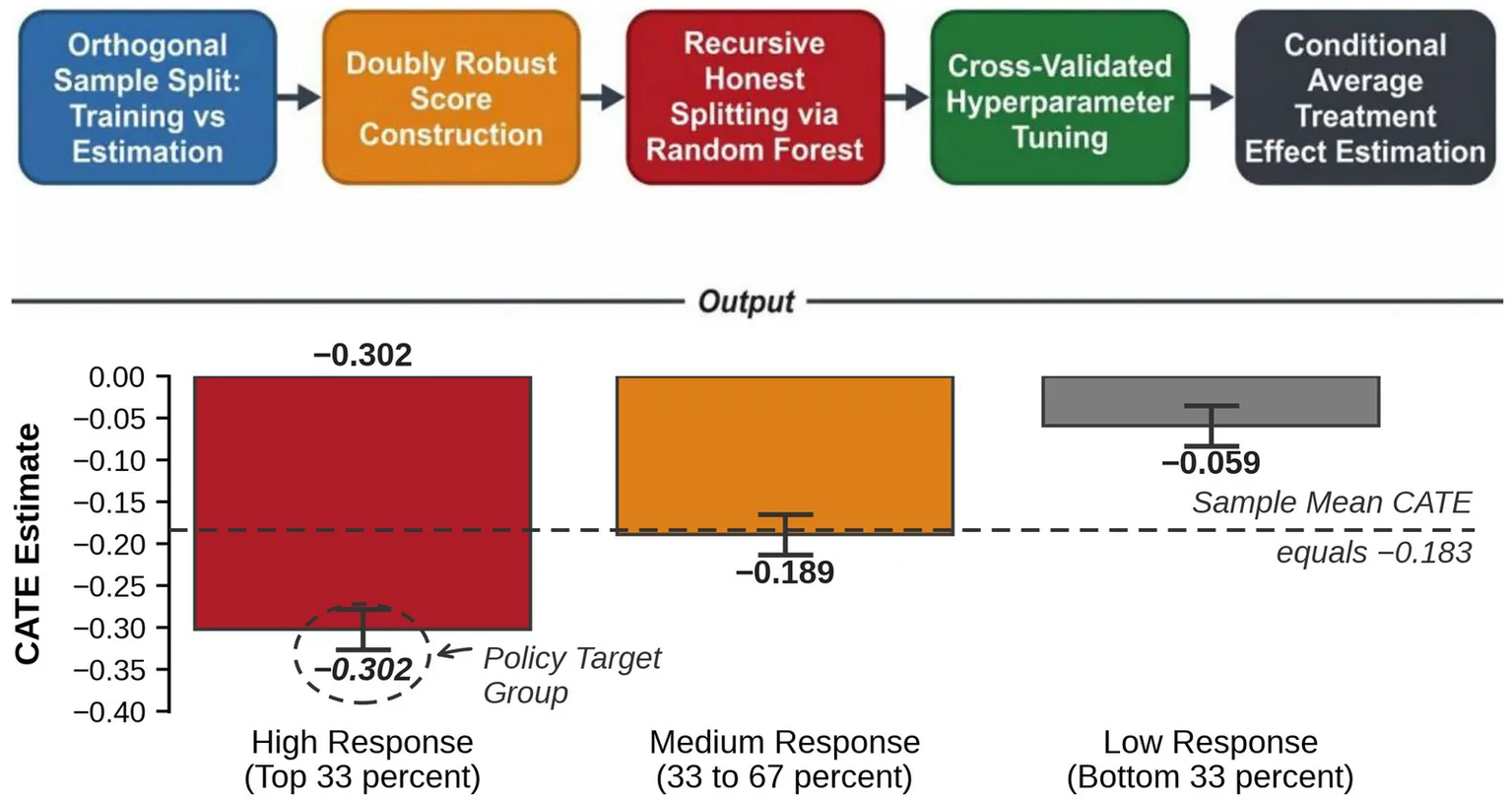
Schematic of causal forest heterogeneity identification and policy heterogeneity grouping.

## 4 Empirical results

### 4.1 Baseline regression results: spillover effects of the three categories of incidents on social trust

After cleaning, matching, and deduplication the sample yields N = 24,576 valid individual by year observations, spanning the four CFPS waves of 2018, 2020, 2022, and 2024 and the merged segment of the four CGSS waves of 2015, 2017, 2018, and 2021, covering respondents aged 18 or above who reported at least occasional attention to competitive sports events and resided in cities within the incident exposure timeline. Descriptive statistics are shown in Table 4. Social trust averages 5.32 and institutional trust 6.41, the established Chinese pattern in which institutional trust exceeds generalized trust. The embedded panel tiers follow the same pattern: the officiating crew and organizing committee is the lowest institutionalized target at 5.16, rising to 6.02 for the sport administration system and 6.38 for public institutions outside sport, while strangers stay at 3.94 and close ties at 8.11, so the largest treatment effects do not arise where baseline trust is already lowest.

**Table 4:** Descriptive statistics of the main variables.

Variable	N	Mean	Standard deviation	Minimum	Maximum
Social trust (0 to 10)	24,576	5.32	2.14	0	10
Institutional trust (0 to 10)	24,576	6.41	1.86	0	10
Trust in strangers (0 to 10)	24,576	3.87	2.29	0	10
Trust in relatives and friends (0 to 10)	24,576	8.17	1.42	1	10
Incident exposure intensity	24,576	0.284	0.371	0	1
Age	24,576	44.63	15.28	18	88
Years of education	24,576	9.31	4.22	0	22
Political interest (0 to 4)	24,576	2.13	1.29	0	4
Media use index	24,576	0.586	0.243	0	1
Urbanization level	24,576	0.634	0.482	0	1
Log household income	24,576	10.87	1.16	6.9	15.2
Trust in the officiating crew and organizing committee (0 to 10)	2,512	5.16	2.31	0	10
Trust in the league and national single sport association (0 to 10)	2,512	5.48	2.18	0	10
Trust in the sport administration system (0 to 10)	2,512	6.02	2.05	0	10
Trust in public institutions outside sport, embedded panel (0 to 10)	2,512	6.38	1.91	0	10
Trust in strangers, embedded panel (0 to 10)	2,512	3.94	2.24	0	10
Trust in close ties, embedded panel (0 to 10)	2,512	8.11	1.47	1	10
Dissatisfaction with the focal incident (0 to 10)	2,512	6.73	2.06	0	10
Perceived institutional signal strength (0 to 10)	2,512	5.87	2.12	0	10
Moral shock (0 to 10)	2,512	6.47	2.27	0	10
Media accessibility index (0 to 1)	2,512	0.612	0.229	0	1
Behavioral transfer share (0 to 1)	2,512	0.436	0.187	0	1

Variables with N = 24,576 are measured on the merged CFPS and CGSS sample; those with N = 2,512 are measured on the balanced embedded panel subsample and averaged across its five analysis waves.

The core estimates of the baseline DID are shown in Table 5. All three categories exert significant negative spillovers on social trust, with procedural incidents largest in absolute terms. With social trust as the dependent variable, the attention weighted ATT of procedural incidents is −0.184 with a standard error of 0.041, about 3.5% of the mean; substantive incidents give −0.098 with a standard error of 0.032 and regulative incidents −0.056 with a standard error of 0.026. On institutional trust the procedural coefficient enlarges to −0.237 with a standard error of 0.048, indicating that trust at the institutional level suffers the deepest damage.

**Table 5:** Baseline regression results of the staggered DID weighted by attention.

Dependent variable	Regulative incidents	Procedural incidents	Substantive incidents	Observations
Social trust	−0.056** (0.026)	−0.184*** (0.041)	−0.098*** (0.032)	24,576
Institutional trust	−0.073** (0.029)	−0.237*** (0.048)	−0.126*** (0.037)	24,576
Trust in strangers	−0.032 (0.028)	−0.117*** (0.038)	−0.061* (0.033)	24,576
Trust in relatives and friends	0.014 (0.019)	−0.028 (0.024)	−0.019 (0.021)	24,576

Robust standard errors clustered at the city level are in parentheses; *, **, and *** denote significance at the 10, 5, and 1% levels respectively; all regressions control for city and year fixed effects as well as basic demographic variables; individual fixed effects are included for the CFPS panel component, and birth cohort fixed effects replace them for the CGSS repeated cross section component.

Trust in relatives and friends shows no significant change under any of the three categories of incidents. Regulative incidents likewise produce no detectable change in trust in strangers, so the pattern of null results is part of the finding rather than a residual: the response is selective and concentrated on institutionalized trust targets rather than evenly distributed across the social sphere. The layered pattern of a stable inner circle and an impacted outer circle matches the surrogate signal framework: diagnostic signals reach institutionalized relations with strangers without disturbing concrete interpersonal trust. The −0.184 effect equals 8.6% of the standard deviation of social trust, in the moderately high range of recent quasi natural experimental studies. The magnitude remains stable within a 3 year window that dilutes the decay of continuous media exposure, so the change is not a transient fluctuation of public opinion; it is, however, confined to specific trust targets and decays after month 24, and is therefore better described as a medium term revision of institutionalized trust than as a lasting reshaping of attitudes. The dynamic effects are shown in Figure 7. Coefficients from 12 months to 1 month before exposure are all insignificant, supporting the parallel trends assumption; the effect emerges in months 2 to 4, peaks at −0.196 in months 7 to 9, and decays after month 24, matching the window specified by Hypothesis H1a.

**Figure 7:**
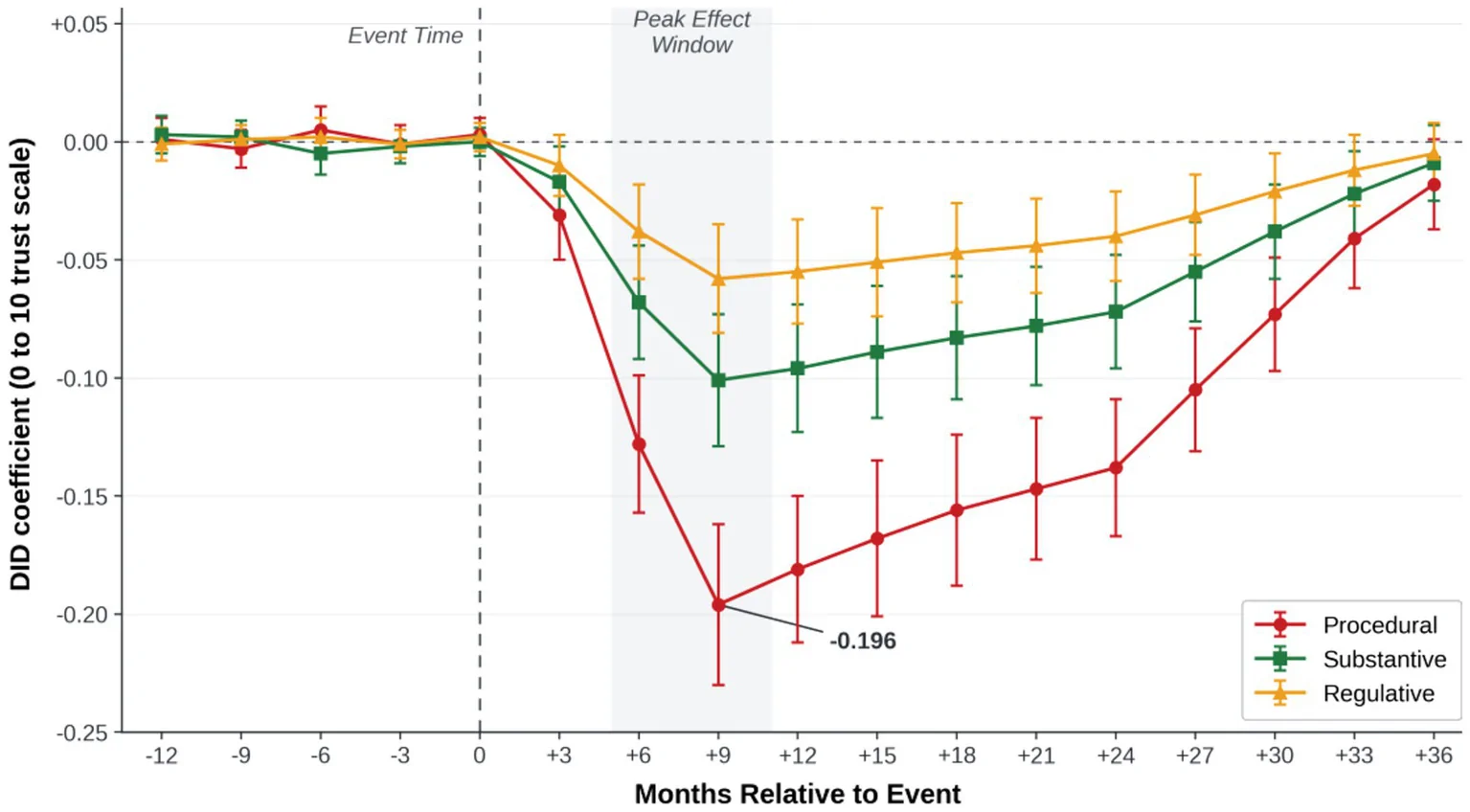
Dynamic effects from the event study (with social trust as the dependent variable).

### 4.2 Effect attenuation along the trust ladder and the boundary of generalization

Whether dissatisfaction with an incident travels beyond the arena is a question about the shape of the effect across trust targets, and it is answered on the embedded panel, where all six tiers are measured for the same individuals and differenced against each respondent’s own pre exposure baseline, with respondents not yet exposed as the comparison group. Baseline tier scores do not differ significantly between respondents subsequently exposed at high and at low intensity, the largest standardized difference being 0.041 with a joint test p value of 0.62. Estimates are reported at the scheduled wave falling between month 8 and month 10 after exposure, inside the peak window identified by the event study, and because the attention weighted aggregate ATT in the survey data is close to the peak of its dynamic path the two sets of estimates are comparable in magnitude. Estimates for procedural incidents attenuate monotonically as institutional distance increases, from −0.612 with a standard error of 0.084 at the officiating crew and organizing committee to −0.221 for public institutions outside sport, which is 36.1% of the innermost effect, and to −0.129 for generalized trust in strangers, while close ties are indistinguishable from zero; the behavioral transfer share moves by 0.048 of a standard deviation. Contrasts between successive segments of the ladder show the decline is not noise: the drop from the officiating crew and organizing committee to the league and national single sport association is 0.218 with a standard error of 0.079, the drop from there to public institutions outside sport is 0.173 with a standard error of 0.071, and the drop from there to close ties is 0.190 with a standard error of 0.068. The three tiers shared with the merged survey data reproduce the survey estimates within one standard error, −0.221 against −0.237, −0.129 against −0.117, and −0.031 against −0.028, so the ladder is not an artefact of the instrument. The full ladder and the generalization test are shown in Table 6.

**Table 6:** Effect attenuation along the trust ladder for procedural incidents, embedded panel.

Tier	Trust target	ATT	Standard error	Share of the innermost tier (%)	Conditional on dissatisfaction with the focal incident
L1	Officiating crew and organizing committee	−0.612***	0.084	100.0	—
L2	League and national single sport association	−0.394***	0.067	64.4	−0.301*** (0.063)
L3	Sport administration system as a whole	−0.286***	0.059	46.7	−0.209*** (0.056)
L4	Public institutions outside sport	−0.221***	0.061	36.1	−0.148** (0.058)
L5	Generalized trust in strangers	−0.129**	0.048	21.1	−0.081* (0.045)
L6	Close ties	−0.031	0.036	5.1	−0.019 (0.034)
—	Behavioral transfer share (standard deviations)	−0.048*	0.025	—	—

Estimates use the balanced subsample of N = 2,512 individuals across the five analysis waves, yielding 12,560 individual by wave observations, differenced against the last scheduled wave before exposure and reported at the wave falling between month 8 and month 10 after exposure. Standard errors clustered at the city level; *, **, and *** denote significance at the 10, 5, and 1% levels. The last column re estimates each tier with dissatisfaction at the incident level and its interaction with exposure as controls, following Equation 5.

Attenuation alone does not settle whether the outer tiers respond to institutional inference or merely carry dissatisfaction about the match watched, so they are re estimated with that dissatisfaction held constant. The effect on public institutions outside sport falls from −0.221 to −0.148 with a standard error of 0.058 and remains significant at the 5% level, retaining 67.0% of the unconditional estimate, and generalized trust in strangers falls from −0.129 to −0.081, retaining 62.8%. About one third of the outer tier response therefore travels with dissatisfaction about the incident itself and about two thirds survives conditioning on it. Because dissatisfaction is measured after exposure, conditioning bounds rather than decomposes the effect, so the share attributable to institutional inference lies between 67.0 and 100% of the unconditional estimate. The regulative and substantive ladders follow the same shape at lower amplitude, with innermost tiers of −0.286 and −0.365 and outside sport effects of −0.068 and −0.118 against survey values of −0.073 and −0.126. The interaction of incident category with ladder tier is shown in Figure 8. Two boundaries follow: close ties do not move under any category, and the study contains no measure of legitimacy beliefs or regime support, so the evidence speaks to the credibility of the bodies that enforce competition rules and, in attenuated form, to public institutions outside sport, not to political delegitimation.

**Figure 8:**
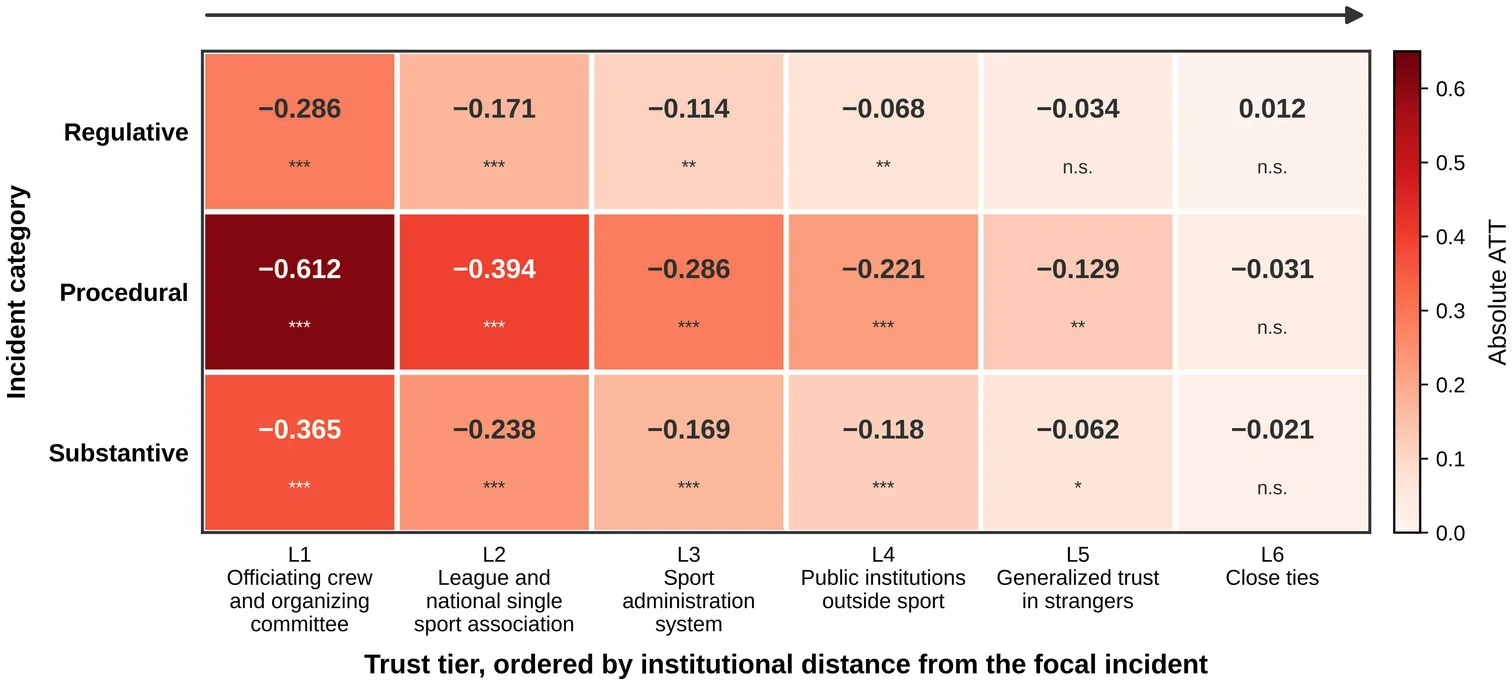
Heat map of the 3 × 6 spillover matrix across incident categories and trust tiers.

### 4.3 Heterogeneity tests across incident types: the dominance of procedural incidents

Effect differences across types address Hypothesis H2. The amplification multiple of procedural relative to regulative incidents is 3.29 and relative to substantive incidents 1.88, with the differences shown in Table 7. The differences involving procedural incidents are statistically significant under Wald tests, whereas the difference between substantive and regulative incidents is not, so the type gradient is established at its upper end and remains indeterminate between the two weaker categories.

**Table 7:** Comparison of effect sizes across the three categories of fairness incidents and significance tests.

Comparison	Coefficient difference	Standard error	Wald statistic	*p* value
Procedural vs. regulative	−0.128	0.039	10.77	0.001
Procedural vs. substantive	−0.086	0.036	5.71	0.017
Substantive vs. regulative	−0.042	0.031	1.84	0.175

The gradient suggests that spectators are most sensitive to the diagnostic signal that “rules can be systematically manipulated,” whereas doping scandals alone, although provoking moral shock, less frequently trigger negative inferences about how rules are enforced beyond the arena. Regulative incidents rank lowest because spectators already hold strong priors about refereeing disputes and a single rule interpretation controversy can hardly update judgments at the institutional level, whereas procedural incidents expose simultaneously that rules can be circumvented and that collusive networks exist, amplifying the signal at both the rule layer and the enforcement layer. The type gradient and the tier gradient are two cuts through the same matrix: procedural incidents dominate at every tier, and the ratio of the procedural to the regulative effect widens from 2.14 at the tier of the officiating crew and the organizing committee to 3.25 at the tier of public institutions outside sport, which indicates that what crosses the boundary of sport is the inference about enforceability rather than the volume of attention an incident attracts. The comparison across incident categories under various combinations of dependent variables and control variables is shown in Figure 9.

**Figure 9:**
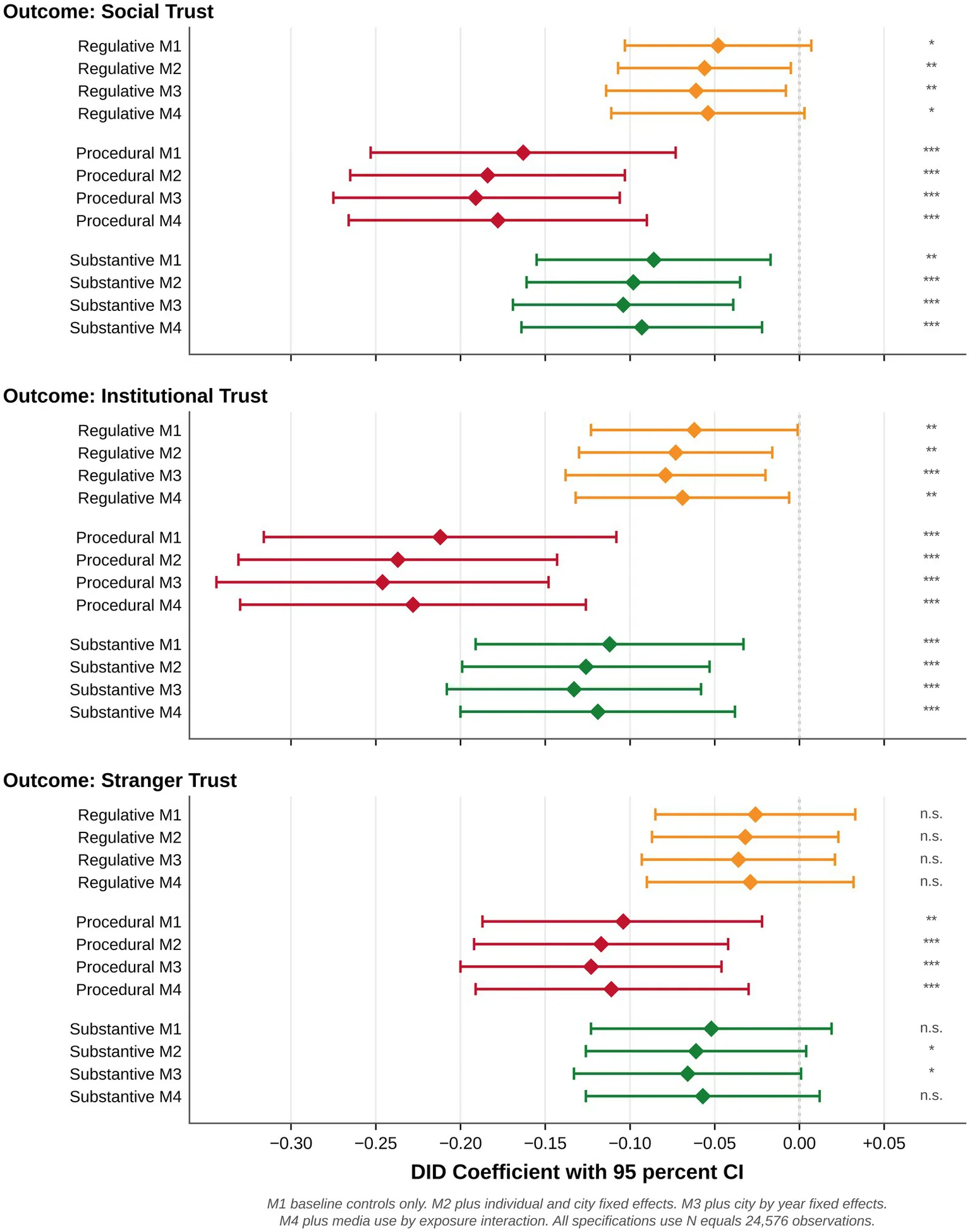
Forest plot of effect sizes for the three categories of incidents.

### 4.4 Synthetic control robustness tests and temporal dynamics

Synthetic control adds counterfactual evidence independent of DID for the procedural estimates. The exposed set comprises the 8 cities at the prefecture level most deeply implicated in the CSL anticorruption investigation from November 2022 to the first half of 2024, identified by hosting a franchise whose officials or players appeared in the publicly released investigation roster, and the donor pool comprises 68 unaffected cities at the prefecture level. The synthetic unit fits the exposed units well before the events, with an RMSPE of 0.058, and path divergence peaks at −0.192 in the ninth month, corroborating the DID estimate of −0.184. Robustness results are shown in Table 8.

**Table 8:** Results of the synthetic control robustness tests.

Test type	Number of treated cities	RMSPE before events	Peak divergence after events	RMSPE ratio (after/before)	Permutation p value
Main analysis	8	0.058	−0.192	2.22	0.028
Replacing the donor pool	8	0.061	−0.186	2.05	0.037
Excluding event years	8	0.056	−0.178	2.14	0.024
Fivefold cross-validation	8	0.063	−0.181	1.94	0.041
Null event placebo	8	0.087	−0.033	0.21	0.386

RMSPE is computed over the 24 months before the events and the 24 months after them. The ratio compares the fit error after the events with the fit error before them, so values above one indicate departure from the synthetic path and values close to or below one indicate none.

Effects at fictitious event dates fail to reject the null at conventional levels, so the identification does not stem from systematic bias in the strategy itself. The ratio of the RMSPE after the events to the RMSPE before them is 2.22 in the main analysis against 0.21 for the null event placebo, and the peak divergence of −0.192 is close to six times the largest deviation of −0.033 obtained at fictitious event dates, so the movement of the actually exposed cities lies outside the range explainable by random fluctuation. The path divergence between the synthetic control and the exposed cities is shown in Figure 10.

**Figure 10:**
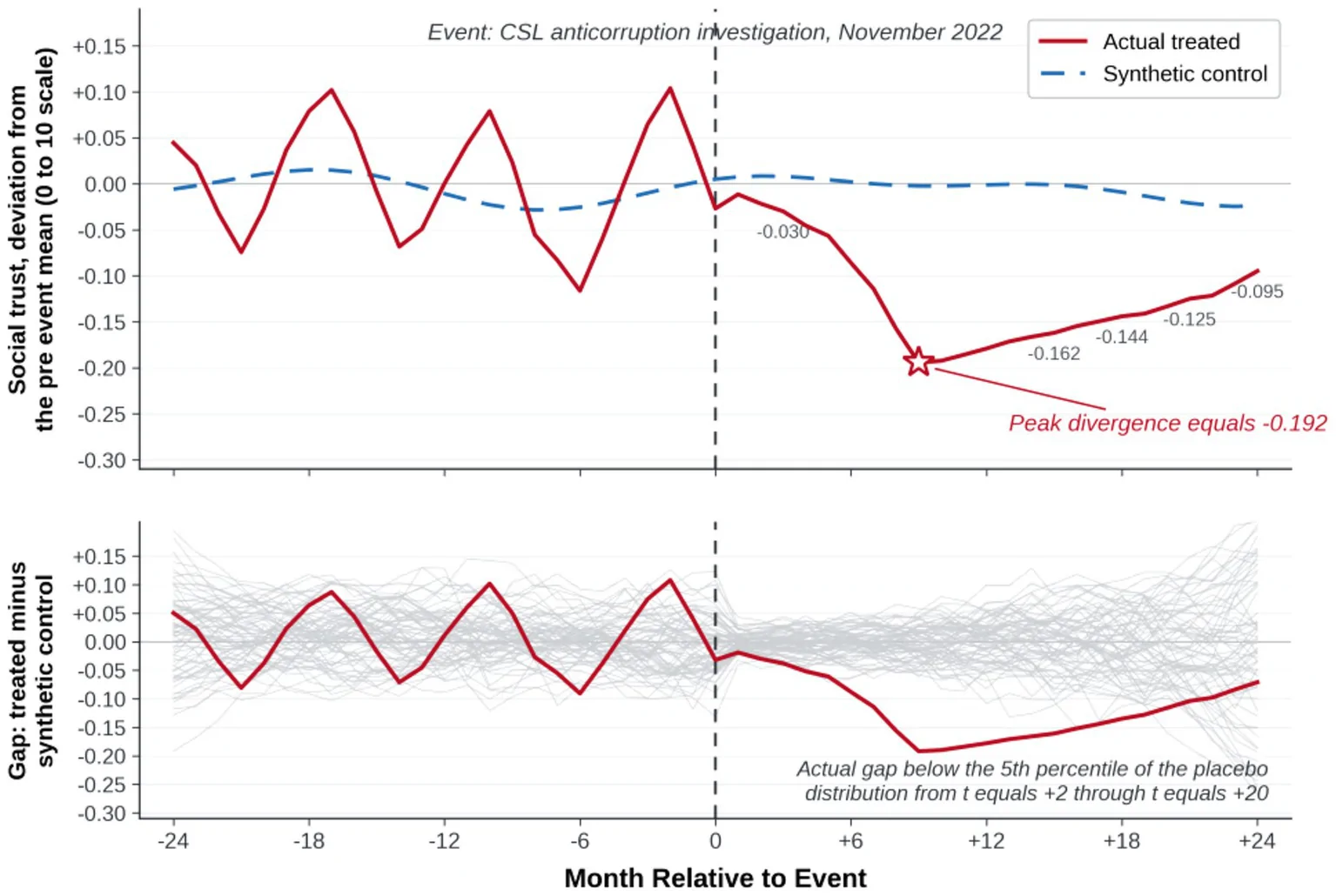
Synthetic control path divergence for the CSL anticorruption investigation.

### 4.5 Competing mechanism decomposition and causal forest heterogeneity identification

The mechanism decomposition responds to Hypothesis H3, with contributions shown in Table 9: the surrogate institutional signal pathway carries 51.3% of the total effect, moral shock 28.4%, and media accessibility 20.3%. The media pathway instrument is robust to strength tests (first stage F = 24.7, far above the Stock-Yogo threshold) and to Anderson-Rubin intervals, and the three contributions remain stable under 500 bootstrap resamples, the largest expansion of standard errors staying below 12% of the baseline.

**Table 9:** Results of the competing mechanism decomposition.

Mediation pathway	Mediation effect	Relative contribution (%)	95% confidence interval	Nullifying correlation
Surrogate institutional signals	−0.094***	51.3	[39.8, 62.7]	0.31
Moral shock	−0.052***	28.4	[18.2, 38.6]	0.24
Media accessibility (IV)	−0.037**	20.3	[8.4, 32.1]	0.19
Total mediated effect	−0.183	100.0	—	—
Direct residual effect	−0.001	—	[−0.024, 0.022]	—

The nullifying correlation is the disturbance correlation between the mediator and outcome equations at which the indirect effect would fall to zero.

Two further checks bear on whether the pathways can be read as mechanisms rather than as correlations among measured constructs. When the contribution shares are re estimated under violations of sequential ignorability, the disturbance correlation at which each indirect effect would fall to zero is 0.31 for the institutional signal pathway, 0.24 for moral shock, and 0.19 for media accessibility, so the dominant pathway is also the least fragile. The pathways further separate on scope as their construct definitions predict: perceived institutional signal strength predicts trust in public institutions outside sport with a standardized coefficient of −0.191 at the 1% level while showing no detectable association with close ties, whereas moral shock predicts the innermost tier strongly at −0.283 and trust outside sport only weakly at −0.058. A mediator carrying generalization should behave in the first way and one carrying event specific affect in the second, and it is on this basis rather than the size of the mediated share that the institutional signal pathway is read as the channel of trust generalization.

The moral shock pathway reaches 41.6% in months 0 to 3, while the surrogate institutional signal pathway rises to 51.3% during months 4 to 12 and peaks at 58.1% during months 13 to 24, the temporal pattern of “emotion first, cognition takes over.” The flow across the three pathways is shown in Figure 11.

**Figure 11:**
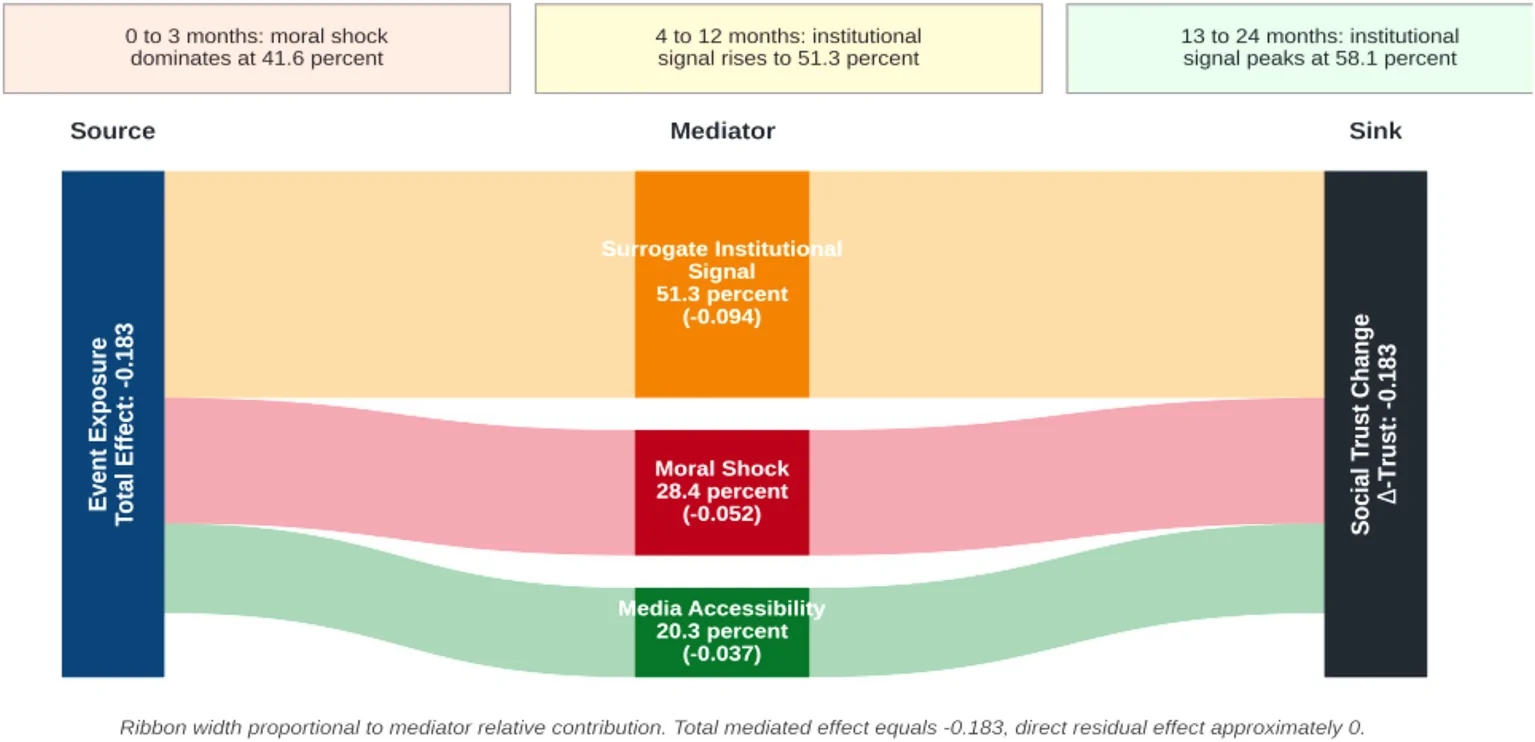
Sankey diagram of the effect decomposition across the competing mechanism pathways.

Causal forest estimation reveals significant effect heterogeneity at the individual level, with a standard deviation of 0.112 and a span of 0.37 between the 5th and the 95th percentile. CATE estimates grouped by core covariates are shown in Table 10.

**Table 10:** Subgroup conditional average treatment effects estimated by the causal forest.

Subgroup	Sample share (%)	CATE estimate	Standard error	Difference from the mean
High political interest (top 25%)	25.0	−0.276	0.048	−0.093
Low political interest (bottom 25%)	25.0	−0.089	0.034	0.094
Urban residents	63.4	−0.212	0.037	−0.029
Rural residents	36.6	−0.128	0.041	0.055
Aged 55 and above	24.8	−0.238	0.052	−0.055
Under age 35	32.3	−0.147	0.043	0.036
High media use (top 25%)	25.0	−0.294	0.056	−0.111
Low media use (bottom 25%)	25.0	−0.096	0.038	0.087

The sample mean of the individual CATEs is −0.183 and differs marginally from the baseline ATT of −0.184 because the causal forest uses honest splitting subsamples.

Political interest and media use are the strongest moderators: the subgroup high in both reaches a CATE of −0.348, which is 1.90 times the full sample mean. The structure of heterogeneity across subgroups is shown in Figure 12.

**Figure 12:**
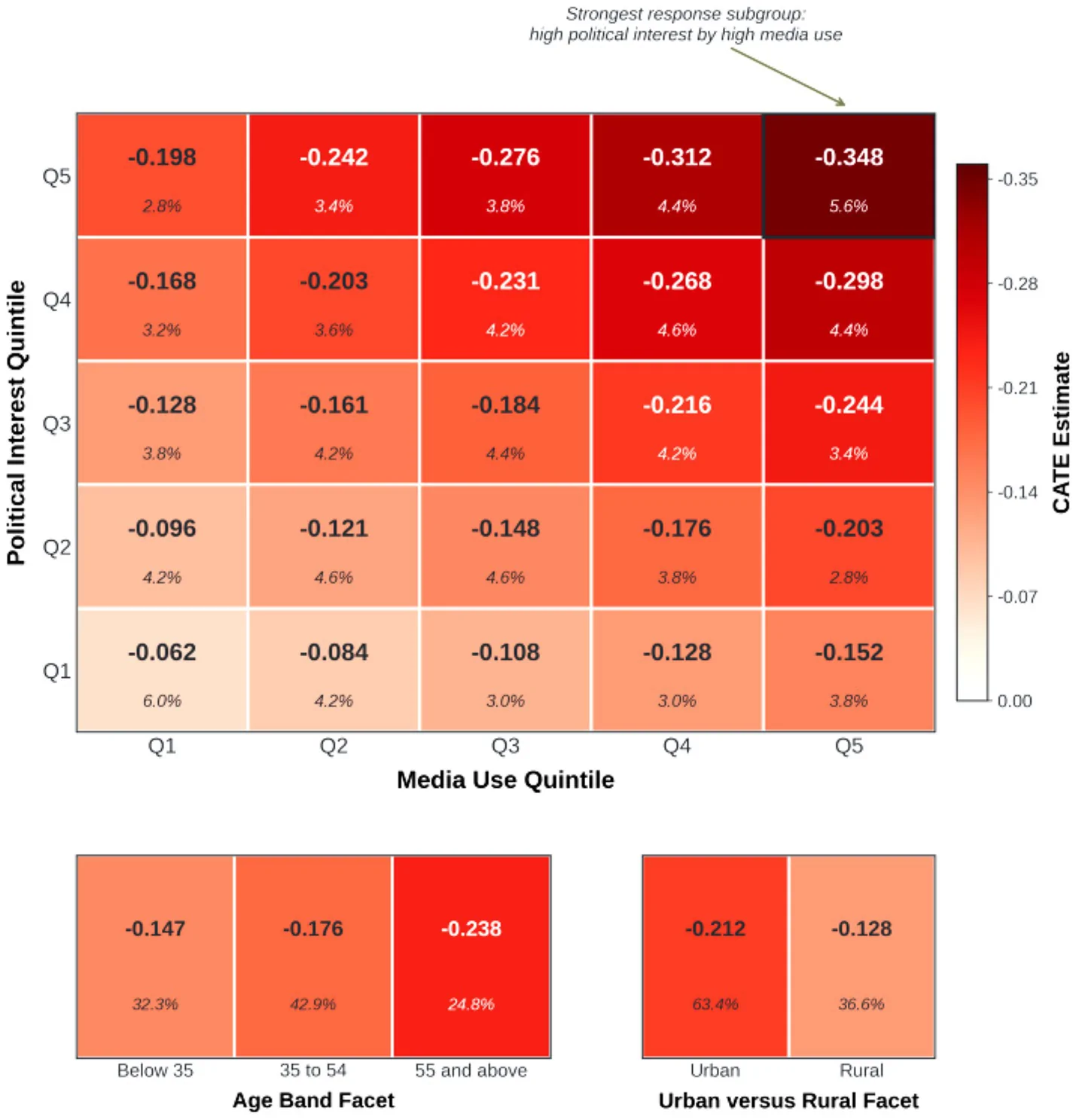
Heat map of CATE heterogeneity across spectator subgroups.

The density of individual CATEs is shown in Figure 13. The distribution is unimodal, peaks at −0.202, and is mildly asymmetric, with a median of −0.189 against a mean of −0.183 and an interquartile range running from −0.261 to −0.112; 14.4% of spectators fall below −0.30 while 2.7% sit above +0.05. Although the average effect is −0.183, this heterogeneity makes any inference that the average represents everyone inaccurate.

**Figure 13:**
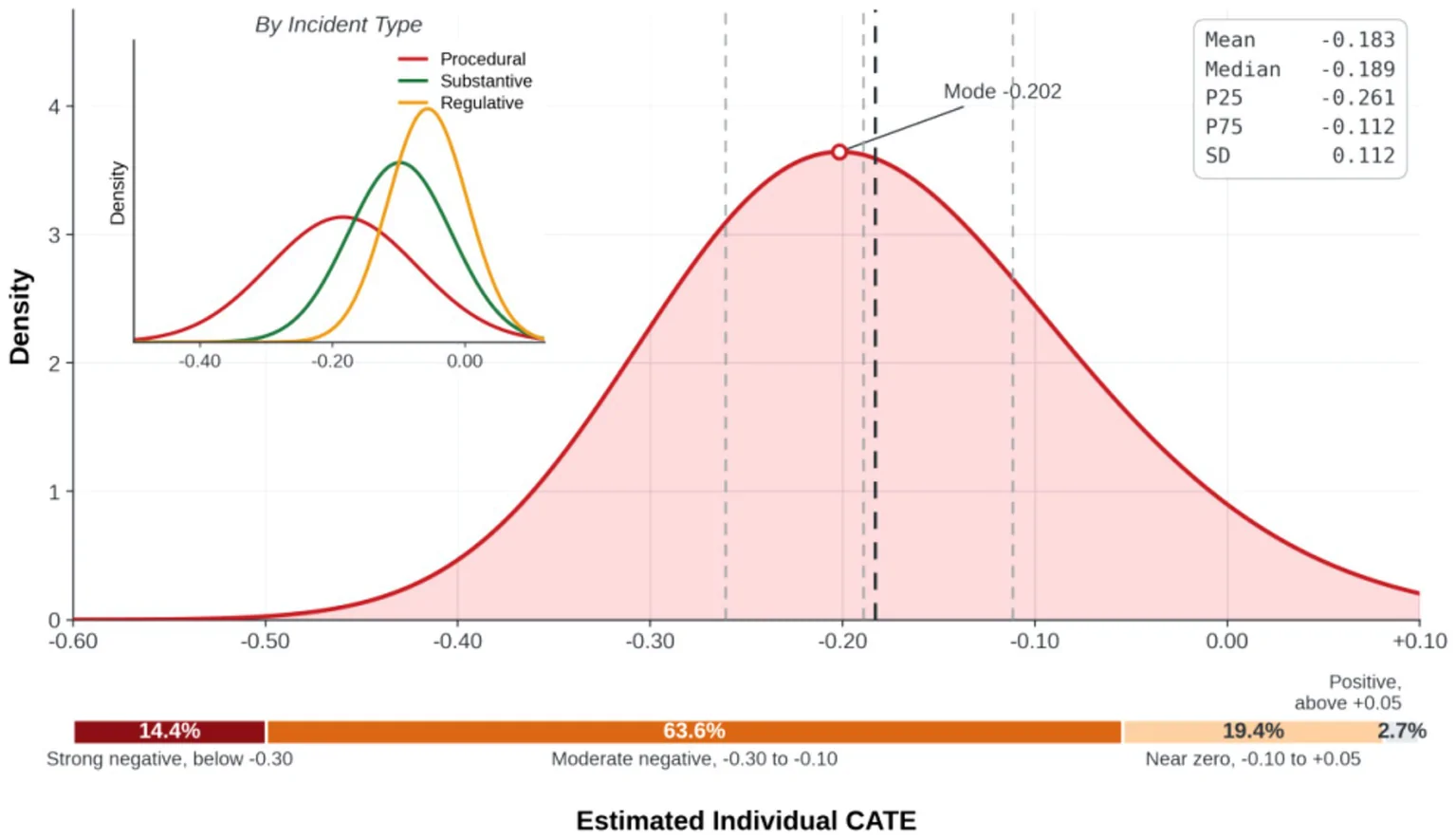
Density plot of the distribution of individual CATEs.

## 5 Discussion and implications

### 5.1 Empirical support for the research hypotheses

The test results for the four propositions are summarized in Table 11, displaying high consistency across methods and across data sources.

**Table 11:** Summary of the empirical test results for the research hypotheses.

Hypothesis	Core proposition	Key evidence	Degree of support
H1a	Incidents exert negative spillovers on social trust, peaking within 6 to 12 months and decaying after 24 months	Aggregate DID ATT of −0.184 with the dynamic path peaking at −0.196, synthetic control peak of −0.192, parallel trends hold	Supported
H1b	The spillover is bounded and graded, attenuating along the trust ladder, with close ties unaffected	Ladder from −0.612 to −0.031, segment contrasts significant, 67.0% retained after conditioning	Supported
H2	Effect gradient of procedural > substantive > regulative, with the effect of procedural incidents strongest	Multiples of 3.29× and 1.88×, Wald p < 0.05 for the procedural comparisons	Supported for the procedural comparisons; not established between regulative and substantive incidents
H3	Surrogate institutional signals dominate, moral shock and media accessibility are secondary, and effects are heterogeneous across spectators	Contributions of 51.3%/28.4%/20.3%, first stage IV F = 24.7, nullifying correlations 0.31/0.24/0.19, CATE 5th to 95th percentile span 0.37	Supported under the stated identification assumptions

Hypothesis H1a obtains dual support from the dynamic DID curve and the synthetic control path divergence: the peak lies in months 7 to 9, matching the expected window of 6 to 12 months, and the effect decays substantially after month 24, so trust updating unfolds over the medium term rather than as transient noise. Hypothesis H1b is confirmed by the shape of the effect across trust targets: the absolute effect declines from −0.612 at the tier of the officiating crew and the organizing committee to −0.221 outside sport and to −0.129 for generalized trust in strangers, close ties do not move, and 67.0% of the outside sport effect survives conditioning on dissatisfaction with the focal incident, so the spillover is bounded and graded rather than diffuse.

Hypothesis H2 is verified by the gradient across categories, with procedural incidents 3.29 times regulative and 1.88 times substantive incidents, and the differences involving procedural incidents statistically significant under Wald tests while the difference between the two weaker categories is not, so the gradient is established at its upper end and left open between regulative and substantive incidents. The gradient confirms that “rules can be systematically manipulated” is the strongest institutional diagnostic signal perceived by spectators.

Hypothesis H3 is confirmed jointly by the three channel decomposition and the causal forest: the pathway contributions are 51.3, 28.4, and 20.3%, the temporal division of labor follows “emotion first, cognition takes over,” and a CATE standard deviation of 0.112 with a span of 0.37 between the 5th and the 95th percentile confirms systematic heterogeneity across subgroups. The pathway result holds under the stated identification assumptions, and the sensitivity thresholds of 0.31, 0.24, and 0.19 indicate how much unmeasured confounding each channel could absorb before its contribution vanished.

The joint validity of the four propositions indicates that the surrogate institutional signal framework has strong explanatory power in the Chinese context: spectators do not treat arena fairness incidents as isolated disputes of entertainment consumption but convert them into belief updates about the reliability of the bodies that enforce the violated rules and, in attenuated form, about public institutions sharing with the arena only the general property of enforcing rules. The reach of the update is finite and measurable, since roughly one third of the effect observed at the arena survives to institutions outside sport and none of it reaches concrete personal ties. The finding pushes institutional trust research, whose observation windows were previously political scandals and corruption cases, toward sports events as a publicly verifiable diagnostic source available at low cost, and the stability of the close ties dimension supplies a clear theoretical boundary that corroborates the classic view that trust is not a unitary construct.

### 5.2 Theoretical contributions and practical implications

The theoretical contributions lie at three levels. Typologically, the three category distinction and the effect gradient show that treating fairness incidents as homogeneous shocks underestimates the strength of trust shocks, and classification by institutional signal supplies new analytical primitives for spillover research. In identification, joining the attention weighted staggered DID with synthetic control, competing mediation, and causal forest diagnostics gives a reusable path “from the average to the distribution,” addressing the bottlenecks of insufficient causal identification and the mechanism black box. At the level of theoretical boundaries, the framework transfers to healthcare, the judiciary, education, and other domains featuring explicit rules, public enforcement, and verifiable outcomes.

These contributions connect to a governance question the Chinese setting poses sharply, namely how fairness, public participation, and high quality sport governance depend on one another. Codified good governance principles place transparency, accountability, and democratic process at the center of federation level reform, and the actors charged with implementing them report that the principles bearing on participation are among the most demanding to put into practice (Anagnostopoulos et al., 2025). Fairness is the component the public can verify without institutional access, because rules, enforcement, and outcomes in competition are observable in real time and by everyone at once. Spectatorship is therefore not passive consumption but the widest available channel of public participation in sport governance: attention supplies a monitoring input at almost no cost, and the fairness of what is observed determines whether that input becomes support or a withdrawal of trust. The results quantify one direction of this loop in the Chinese context: when the observable component of governance quality fails, the monitoring channel transmits the failure outward at a measurable rate, retaining about one third of its initial magnitude at institutions outside sport, and stopping at the boundary of institutionalized trust. High quality sport governance is therefore not only an internal management objective but a condition for preserving the credibility on which the participation channel depends.

The practical implications point to coordinated responses among the authorities implicated by an arena incident and the media that carry it. In the Chinese configuration these authorities are three distinguishable sets of actors. The first is the rule owning bodies, namely the professional league companies and the national single sport associations, which stage the competition, own the rules and the disciplinary procedures, and characterize a case in the first instance; the event organizers discussed below belong to this set. The second is the sport administration departments, namely the General Administration of Sport of China and its provincial and municipal counterparts, which hold policy, funding, and personnel authority over those bodies. The third is the supervisory and judicial organs, whose entry converts a disputed incident into an officially characterized one and which determine liability once conduct crosses disciplinary or criminal thresholds. The response strategies of the first two sets should revolve around the transmission logic of surrogate signals: institutional diagnostic signals are the dominant pathway, so timely, transparent, and verifiable official disclosure supplies reverse diagnostic signals and has the leverage to weaken negative spillovers, with the speed and the substantive content of information governance as the joint conditions for trust repair. Because the effect is largest at the tier of the bodies that own the violated rules, disclosure issued by those bodies acts where the damage is concentrated, whereas the supervisory and judicial organs arrive later and shape the credibility rather than the speed of the response; the decade long reform record indicates that periodic rectification campaigns raise disclosure mainly for the duration of the campaign, consistent with the finding that the effect decays rather than being actively repaired (Li et al., 2025). As rule owning bodies, event organizers should embed typological screening into their internal risk management systems, placing procedural risks such as collective deliberate underperformance and illicit benefit transfers under the highest priority compliance monitoring rather than focusing only on technical refereeing disputes. Media accessibility still contributes one fifth of the total effect, so inflammatory reporting chasing clicks amplifies the public cost of the trust gap, whereas credible independent disclosure channels at critical junctures compress the transmission window of surrogate signals.

### 5.3 Scope conditions and the limits of the evidence

Setting the boundary of a claim is part of stating it, and three distinctions carry the interpretation. Dissatisfaction with a particular incident is the largest and least surprising response, measured directly rather than inferred from more distant tiers. Trust in the bodies that own the violated rules is the tier at which the framework does its substantive work, retaining close to two thirds of the innermost effect. Trust in public institutions outside sport is the tier at which the term spillover is warranted, retaining 36.1% of the innermost effect, of which 67.0% survives conditioning on dissatisfaction with the focal incident, a figure bounding the generalized component from below. Beyond that tier the evidence thins quickly: generalized trust in strangers moves by about one fifth of the innermost effect, significantly for procedural incidents, marginally for substantive incidents, and not detectably for regulative incidents, and close ties do not move at all. The claims are set against the supporting evidence in Table 12.

**Table 12:** Claims, supporting evidence, and scope boundaries.

Claim	Supporting evidence	Status
Fairness incidents reduce trust in the officiating crew and organizing committee	L1 estimate of −0.612 (0.084)	Established
The effect extends to the league, the national association, and the sport administration system	L2 and L3 of −0.394 and −0.286, contrast with L1 of 0.218 (0.079)	Established
A fraction of the effect reaches public institutions outside sport	L4 of −0.221, 36.1% of L1, 67.0% retained after conditioning	Established with the stated attenuation
The effect reaches generalized trust in strangers	L5 of −0.129, 21.1% of L1, significant at 5% for procedural and 10% for substantive incidents	Weak and category specific
The effect reaches trust in close ties	L6 of −0.031, not significant under any category	Not supported
The effect constitutes institutional delegitimation or a political shock	No measure of legitimacy beliefs or regime support; effect decays after month 24	Outside the scope of this study

Two readings are therefore not available. The first is institutional delegitimation: a decline of 0.221 points on a 0 to 10 scale that decays after month 24 is a revision of an evaluation rather than a withdrawal of legitimacy, and neither legitimacy beliefs nor regime support is measured here. The second is political spillover in the strict sense: the outer tier consists of courts, local administrative departments, and public service institutions, asked about as providers of rule bound public services, and no item in the three data sources refers to political institutions. What the results establish is narrower and more usable: a fairness failure in a highly observed arena imposes a measurable and time limited cost on the credibility of the bodies that enforce competition rules, a defined fraction transfers to institutions sharing only the property of enforcing rules, and the transfer stops before reaching the concrete relationships on which everyday cooperation rests.

## 6 Conclusion

Based on the surrogate institutional signal framework, this study of the spillover effects of fairness incidents in sports events on spectators’ social trust reaches the following core conclusions:

(1) Fairness incidents in sports events reduce spectators’ trust in the bodies implicated by the incident and, in attenuated form, in public institutions outside sport; the attention weighted effect on social trust is approximately 3.5% of the mean, peaks in months 7 to 9 after incident exposure, and gradually decays after 24 months.
(2) The spillover is graded rather than diffuse: the absolute effect falls from −0.612 at the tier of the officiating crew and the organizing committee to −0.221 at the tier of public institutions outside sport and to −0.129 for generalized trust in strangers, and 67.0% of the effect at the tier outside sport survives conditioning on dissatisfaction with the focal incident.
(3) The effects of the three categories of incidents display a systematic gradient; the absolute effect of procedural incidents is 3.29 times that of regulative incidents and 1.88 times that of substantive incidents, confirming that “rules can be systematically manipulated” constitutes the strongest institutional diagnostic signal.
(4) Surrogate institutional signals are the dominant transmission pathway, carrying 51.3% of the total spillover effect, while moral shock and media accessibility contribute 28.4 and 20.3% respectively, presenting the temporal evolution of “emotion first, cognition takes over.”
(5) Effects are significantly heterogeneous across spectator subgroups; the CATE of the intersecting subgroup with high political interest and high media use reaches −0.348, which is 1.90 times the full sample mean.
(6) The stability of the trust in relatives and friends dimension reveals that trust is a layered, multidimensional construct; institutional diagnostic signals spill over only to institutionalized relations with strangers without disturbing established concrete interpersonal trust, and no measure in this study bears on legitimacy beliefs or regime support.

The limitations lie mainly in an incident sample centered on the Chinese context, so external validity across cultures awaits testing, and in the technical constraints of aligning the three data sources temporally at the individual level. The mechanism decomposition further rests on assumptions about unmeasured confounding that sensitivity analysis can bound but not remove, and incident identification retains official characterization as an entry condition, which excludes never characterized incidents and may understate the frequency of the regulative category. The inner tiers of the ladder are measured on an incentivized online panel rather than a probability sample, so the levels observed there should not be read as nationally representative, although the estimated changes are differenced against each respondent’s own pre exposure baseline and reproduce the survey estimates at the shared tiers. Future research can extend to healthcare, the judiciary, and other institutional domains with public diagnostic signals, and introduce higher frequency real time trust measures to characterize the switching point between the emotional and the cognitive pathway more finely.

## Data Availability

Publicly available datasets were analyzed in this study. The CFPS and CGSS microdata can be obtained upon registration from the Institute of Social Science Survey, Peking University, and the National Survey Research Center, Renmin University of China, respectively. The embedded panel data and the incident database, together with the codebook, the operational coding rules, the adjudication records, and the item wording for the six trust tiers and the three mediators, will be made available by the authors, without undue reservation.
